# Modifications of EHPDB Physical Properties through Doping with Fe_2_O_3_ Nanoparticles (Part II)

**DOI:** 10.3390/ijms23010050

**Published:** 2021-12-21

**Authors:** Sebastian Lalik, Olaf Stefańczyk, Natalia Górska, Kunal Kumar, Shin-ichi Ohkoshi, Monika Marzec

**Affiliations:** 1 Faculty of Physics, Astronomy and Applied Computer Science, Institute of Physics, Jagiellonian University, 30-348 Kraków, Poland; sebastian.lalik@doctoral.uj.edu.pl; 2 Department of Chemistry, School of Science, The University of Tokyo, Bunkyo-ku, Tokyo 113-0033, Japan; olaf@chem.s.u-tokyo.ac.jp (O.S.); kunal-k@chem.s.u-tokyo.ac.jp (K.K.); ohkoshi@chem.s.u-tokyo.ac.jp (S.-i.O.); 3 Faculty of Chemistry, Jagiellonian University, 30-387 Kraków, Poland; natalia.gorska@uj.edu.pl

**Keywords:** ferroelectric liquid crystal, *γ*-Fe_2_O_3_ nanoparticles, light absorption, dielectric relaxation, magnetization, metal-organic nanocomposite

## Abstract

The aim of our study was to analyze the influence of various concentrations of *γ*-Fe_2_O_3_ nanoparticles on the physical properties of the liquid crystalline ferroelectric SmC* phase, as well as to check the effect of introducing nanoparticles in the LC matrix on their properties in the prepared five nanocomposites. UV-vis spectroscopy showed that the admixture reduced the absorption of nanocomposites in the UV range, additional absorption bands appeared, and all nanocomposites were transparent in the range of 500–850 nm. The molecular dynamics in particular phases of the nanocomposites were investigated by the dielectric spectroscopy method, and it was found that nanoparticles caused a significant increase in the dielectric constant at low frequencies, a strong modification of the dielectric processes in the SmC* phase, and the emergence of new relaxation processes for the highest dopant concentrations. SQUID magnetometry allowed us to determine the magnetic nature of the nanoparticles used, and to show that the blocked state of nanoparticles was preserved in nanocomposites (hysteresis loops were also registered in the ferroelectric SmC* phase). The dependence of the coercive field on the admixture concentration and the widening of the hysteresis loop in nanocomposites in relation to pure nanoparticles were also found. In turn, the FT-MIR spectroscopy method was used to check the influence of the impurity concentration on the formation/disappearance or modification of the absorption bands, and the modification of both the FWHM and the maximum positions for the four selected vibrations in the MIR range, as well as the discontinuous behavior of these parameters at the phase transitions, were found.

## 1. Introduction

Hybrid materials are of great interest in the modern world because they can simultaneously exhibit many interesting properties. Typically, hybrid materials are materials that result from the combination of inorganic and organic chemical objects. In the soft matter field, hybrid materials can be created based on the combination of, e.g., a liquid crystal (compound or binary, three or more component mixture) and nanomaterial (nanoparticles, nanotubes, quantum dots, or nanorods). The huge variety of both liquid crystal materials and nanomaterials allows the creation of a number of hybrid materials with interesting properties. However, liquid crystals have been known since 1888, and only recent nanotechnology development has opened up new research opportunities. Generally, the synthesis of liquid crystals from a chemical point of view is very complex, multistep, costly, and time-consuming, even within one homologue series, and the parameters of synthesized materials are usually not adjusted to a specific application. Thus, in order to tune the parameters (phase transition temperatures, spontaneous polarization, threshold voltage, switching time, molecular tilt angle, extent of light transmission, light absorption range, dielectric permittivity, impedance and conductivity, layer roughness, stiffness, etc.), scientists modify the molecular structure. Such modifications cover, for example: adding more aromatic parts to the rigid core, increasing the number of methylene groups, fluorination of the side chain and/or aromatic parts, and introduction of heteroatoms or polymerization reactions in order to obtain polymer dispersed liquid crystals (PDLCs) [1,2,3,4,5,6,7,8,9,10].

It turns out that many interesting parameters of liquid crystalline materials can be modified by doping with nanomaterials. This path is quite simple, because by changing the concentration of the nanomaterial, it is possible to obtain modified properties of the final nanocomposite, and at the same time, such a hybrid material combines the features of both constituent materials. Additionally, nanomaterials are easily available on the market, and their low cost favors this method. Despite many advantages, nanocomposites based on liquid crystals are still not very popular, and so far, most of the published articles have focused on nanocomposites based on nematics. Huang et al. observed an increase in photoluminescence for the concentration of Ag nanoparticles (ca. 88 nm) up to 1 wt %, then its decrease (up to 4 wt %) in the nanocomposite based on the liquid crystal 5CB, which was explained by the surface plasmon resonance for nanoparticles [11]. Magnetic needles were used to enriched nematic liquid crystal, and as a result, the planar alignment from the homeotropic one was obtained in low magnetic fields (10–30 G) [12]. Zadoina et al. showed that after doping liquid crystal polymer by nanomagnets (cobalt nanorods, ca. 5.5 nm, length 83 nm, 4 wt %) the liquid crystal order was preserved, T_g_ decreased, and the magnetic properties and the nanorods’ orientation were improved [13]. In turn, the decrease in the threshold voltage was observed after doping 6OCB by Fe nanoparticles (ca. 35–45 nm, 0.25 wt %) [14], as well as after addition of BaTiO_3_ nanoparticles (ca. 50 nm, 0.5 wt %) into a polymer-dispersed liquid crystal based on E7 [15]. Interestingly, Hasegawa et al., after doping nematic liquid crystal by BaTiO_3_ nanoparticles (ca. 80 nm, 0.25 wt %), decreased the T_NI_ transition temperature without modification of the threshold voltage [16]. It turned out that after addition of *γ*-Fe_2_O_3_ nanoparticles (needles, diameter ca. 25 nm, length 175 nm, 0.05 wt %, decorated by various surfactants) into 5CB liquid crystal, the molecular structure of the surfactant affected the nematic order, as well as nanoparticles’ aggregation process [17]. By doping nematic liquid crystal EBBA with metallic multiwalled carbon nanotubes (MWCNT, diameter ca. 12–20 nm, length several micrometers, up to 1.0 wt %), a conductive network was formed in an organic matrix, and for a nanocomposite with 0.15 wt % MWCNT, the electric conductivity increased by about two orders of magnitude [18]. Shriyan et al. also used MWCNT as dopant into PDLC, and observed decreasing of the resistivity and increasing of the capacitance, especially for high admixture concentrations; while for small concentrations, the rise and fall times were improved, which was explained by smaller liquid crystal droplets in the polymer matrix [19]. In turn, by doping 8OCB with Fe_2_O_3_ nanoparticles (ca. 6–12 nm, 0.5 and 1.0 wt %), better alignment of molecules, fewer defects, an increase in dielectric dispersion and in V_th_, as well as a decrease in the dielectric anisotropy, was obtained. The shift of the relaxation process towards a lower frequency was explained by the different surface potentials and interactions between LC molecules and nanoparticles [20].

Despite many promising features of smectic liquid crystals, in the literature there are only a few articles about doping them. This is what motivated our research. The most interesting of the smectic phases is the ferroelectric SmC* phase built of chiral molecules. In this phase, the organic molecules are arranged in layers and tilted (in a different direction at each layer, forming a helix of the resultant dipole moments). It is known that the spontaneous polarization appears under the external electric/magnetic field in this phase [21]. The strong-enough external field causes the reorientation of the dipole moments along the field lines, and thus unwinding of the helix (all dipoles are aligned with the external field, and the spontaneous polarization reaches its maximum) [22]. The use of an alternate electric field changes the orientation of the macroscopic polarization, in what is known as electro-optic switching (one of the most important utility parameters of these materials) [23,24]. If there is more than one polar group in the structure of an organic molecule and their distance from the chiral center is small, then the dipole moment of such a molecule is greater, and therefore the spontaneous polarization is higher. Three years after the discovery of ferroelectric liquid crystal (FLC), the existence of a periodic structure (helix) in the SmC* phase was confirmed by Yoshino et al., who observed a periodic pattern in polarized light, and a change in the intensity of light passing through a thin FLC layer as the applied voltage changed. For the unwound helix, a decrease in light scattering and thus an increase in its transmission was observed [25]. Prakash et al. showed that spontaneous polarization and rotational viscosity increased, while dielectric loss factor was an order of magnitude larger after doping the ferroelectric liquid crystal LAHS18 with MWCNTs (ca. 30–50 nm, length > 0.3 μm, 0.1 wt %). This behavior was explained by the increase in electric conductivity and the formation of a conducting network through nanotubes. Interestingly, a nonzero P_s_ was observed in the SmA* phase, which was attributed to the possible existence of a short-range orientational order, caused by, inter alia, surface anchoring organic molecules to MWCNTs and ionic impurities in the doped sample [26]. Two ferroelectric liquid crystals (Felix 17/000, Felix 16/100) were doped with quantum dots of Cd_1−x_Zn_x_S/ZnS (ca. 8.7 nm, 1.0 wt %) and CdSe (ca. 3.5 nm, 1.0 wt %), and it was found that the tilt angle was smaller by about 8° (as an attraction effect between quantum dots surrounding the molecules), and P_s_ decreased (as the secondary order parameter coupling with the tilt angle) for the doped Felix 17/000. Moreover, a new relaxation process around 10^5^ Hz was revealed for the doped Felix 16/100 [27]. Das et al., after addition of Au nanoparticles (ca. 6–8 nm) to nonchiral liquid crystal with an SmC phase, observed a decrease in all phase transition temperatures, a strong increase in light absorption for two bands below 300 nm, and a 4-fold increase in electric conductivity in the crystal phase [28]. Dey et al. used the antiferroelectric liquid crystal DM1 as a host for MWCNTs (ca. 8–15 nm, length 0.5–2.0 μm, 0.12 wt %) and obtained better alignment (more homogeneous textures), while the primary order parameter was not disturbed and the rotational viscosity and switching time decreased by about 50%, and the electric conductivity decreased (as a result of trapping ions). Interestingly, the PL (in-phase fluctuations of the director’s phase in adjacent layers) relaxation mode was not observed, while the relaxation frequency of the PH (anti-phase fluctuations of the director’s phase in adjacent layers) mode drastically decreased in the SmC*_A_ of the doped sample. Moreover, the Goldstone mode in the antiferroelectric SmC*_A_ was hereditary from the SmC* phase, and a new relaxation process was revealed after suppression of the Goldstone mode, but only for the doped sample [29]. Chaudhary et al. doped an FLC W206E mixture with ZnO nanoparticles (ca. 10 nm, 0.1–1.0 wt %) and observed the memory effect (explained by charge transfer from the organic molecule to the ZnO nanoparticle) and more centers to the charge transfer for higher concentrations [30]. In turn, after doping the ferroelectric mixture ZLI-4851 with Ni nanoparticles (ca. 20 and 40 nm, 0.5 wt %) an increase in the spontaneous polarization and a shortening of the switching times of about 200 μs were observed [31]. In turn, the influence of carbon nanomaterials on the nematic and smectic liquid crystals can be found in review papers [32,33]. Joshi et al. showed that Al_2_O_3_ (ca. 4–7, 20–30, 30–50 nm) admixed with KCFLC 7S and 10S caused the adsorption of impurities to their surfaces [34]. Such adsorption of impurities to the nanoparticle surface was also observed by Chandran et al. when doping KCFLC 10S with ZrO_2_ nanoparticles (ca. 30 nm, 4 wt %) [35]. Singh et al. doped Felix 16/100 with a ZnO_1−x_S_x_ nanomaterial (ca. 10–15 nm, 0.25, 0.50, 1.0 and 1.5 wt %), and explained that the observed influence of nanoparticles on the dielectric increment and relaxation frequency was related to the formation (by agglomerates) of zigzag defects or a chevron structure [36]. Singh et al. reported that one of the effects of doping CdSe quantum dots (QDs, ca. 3.5 nm, 0.5, 1.0 and 2.0 wt %) onto Felix 16/100 was the emergence of a new relaxation mode (attributed to dipolar interactions between FLC molecules and quantum dots) depending on their concentration, as well as the modification of the GM relaxation frequency [37]. In turn, the new relaxation process observed by doping Felix 17/100 with ZnS QDs was attributed to the flexoelectric tilt fluctuations in the FLC next to QDs [38]. Finally, it should be noted that the excess of nanoparticles in the created nanocomposites may destroy or strongly disturb the liquid crystal order, which in turn may lead to the uselessness of the created hybrid materials.

The aim of our work was to show how *γ*-Fe_2_O_3_ nanoparticles affected the physical properties of the EHPDB liquid crystal. The created nanocomposites consisted of an organic matrix (liquid crystal EHPDB) exhibiting ferroelectric properties, and an inorganic guest (*γ*-Fe_2_O_3_ nanoparticles) exhibiting a paramagnetic and blocked state. In our previous paper, we presented in detail the synthesis of studied nanocomposites and results of diffraction, microscopic, calorimetric, electric and FT-MIR spectroscopy methods, and showed that modification of the electric parameters of the EHPDB liquid crystal by nanoparticles was significant; however, to explain this further, research is needed [39]. Therefore, in this paper we decided to examine how interactions between components of the composites were revealed in their properties by using light absorption in the UV-vis and MIR (FT-MIR) range, frequency domain dielectric spectroscopy (FDDS), and magnetic superconducting quantum interference device magnetometry (SQUID) methods. Influence of the temperature and nanoparticle concentration, as well as oleic acid (used to nanoparticles decoration), on the intensities of the chosen absorption bands were carefully analyzed. The modification of the specific electric conductivity by nanoparticles and their influence on the parameters of relaxation processes (Δε, ν_r_, τ_r_, α, σ_DC_) were also investigated. In turn, SQUID was used to determine the blocking temperature, T_B_, of nanoparticles based on ZFC (zero field cooled) and FC (field cooled) curves, and to examine the influence of nanoparticles on the magnetization, hysteresis loops, the coercive field, and the remanence. A detailed analysis of the absorption bands in the middle infrared region was also performed. We believe that by easily modifying the parameters of the SmC* phase with small nanoparticle concentrations, such nanocomposites will find application in industry or medicine, where nematic materials currently dominate [40], or will be a good alternative to orthoconic liquid crystals by obtaining good dark states for a smaller voltage threshold [41].

## 2. Results and Discussion

### 2.1. Samples Studied

All created nanocomposites were metal–organic hybrid systems with ferroelectric liquid crystal EHPDB as the matrix (host) and *γ*-Fe_2_O_3_ nanoparticles as an admixture (guest). Properties of six nanocomposites denoted as Composites 1–6 (Composite 1 (0.0 wt %), Composite 2 (0.3 wt %), Composite 3 (0.5 wt %), Composite 4 (0.7 wt %), Composite 5 (0.9 wt %) and Composite 6 (0.3 wt % + oleic acid)), as well as pure nanoparticles of *γ*-Fe_2_O_3_ (Sample 1) and nanoparticles decorated with oleic acid (Sample 2), were studied. A detailed description of the sample preparation can be found in our recent paper [39], while phase sequence during cooling of the prepared composites was as follows (phase transition temperatures in parentheses are in Celsius):

Composite 1: Is(96.29)P(95.11)N*(83.19)TGBA*(~80.06)SmA*(~79.00)SmC*(58.07)Cr_1_
Composite 2: Is(91.24)P(?)N*(79.42)TGBA*(?)SmA*(72.20)SmC*(55.79)Cr_1_
Composite 3: Is(89.10)N*(79.85)TGBA*(?)SmA*(67.70)SmC*(53.48)Cr_1_
Composite 4: Is(89.63)P(?)N*(78.51)TGBA*(?)SmA*(69.50)SmC*(54.61)Cr_1_
Composite 5: Is(91.91)N*(80.09)TGBA*(?)SmA*(69.80)SmC*(55.56)Cr_1_
Composite 6: Is(73.21)X(72.09)N*(62.64)SmA*(52.36)SmC*(50.76)Cr_1_(39.84)Cr_2_

A question mark in parentheses means that the transition temperature could not be determined (or was subject to a very large error), while P denotes the blue phase.

### 2.2. Ultraviolet-Visible Spectroscopy (UV-Vis) Results

#### 2.2.1. UV-Vis Results for Samples 1–2

**Figure 1:**
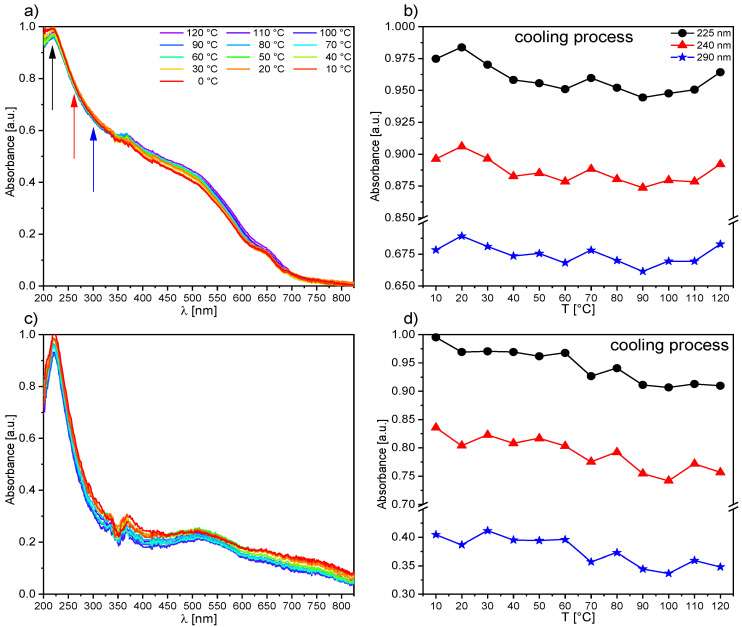
The solid state UV-vis absorption spectra for Sample 1 (**a**) and Sample 2 (**c**) in the 200–850 nm range, as well as the temperature influence on the chosen wavelengths for Sample 1 (**b**) and Sample 2 (**d**). The legend in graph (**a**) is the same for (**c**), while that in graph (**b**) is the same for graph (**d**).

Both Sample 1 and Sample 2 absorbed in the 200–700 nm range (brown color of maghemite *γ*-Fe_2_O_3_ nanoparticles [42]) with clear absorption bands around 220, 370, 500, and 650 nm (Figure 1a,c). Intensity of absorption was much lower for Sample 2 than for Sample 1 (between 250 nm and ca. 600 nm). In our opinion, this could be due to the fact that Sample 2 contained fewer *γ*-Fe_2_O_3_ nanoparticles, which in addition were immersed in oleic acid. It is possible that oleic acid, in measuring the wavelength range, was transparent, so it did not absorb. It was noteworthy that absorption of indicated wavelengths (e.g., 225, 240, and 290 nm, marked by arrows in Figure 1a) behaved the same for both samples (oscillated), and did not change significantly upon cooling from 120 °C down to 0 °C (Figure 1b,d), although the absorbance at the wavelengths under consideration was much lower after the addition of oleic acid. Interestingly, at 225 nm, Sample 1 (pure nanoparticles), as well as Sample 2 (with oleic acid), absorbed almost all the incident radiation, while the absorption band width with a maximum of around 220 nm was narrower for Sample 2. In summary, the oleic acid significantly modified the optical properties of the nanoparticles used.

#### 2.2.2. UV-Vis Results for Composite 1

**Figure 2:**
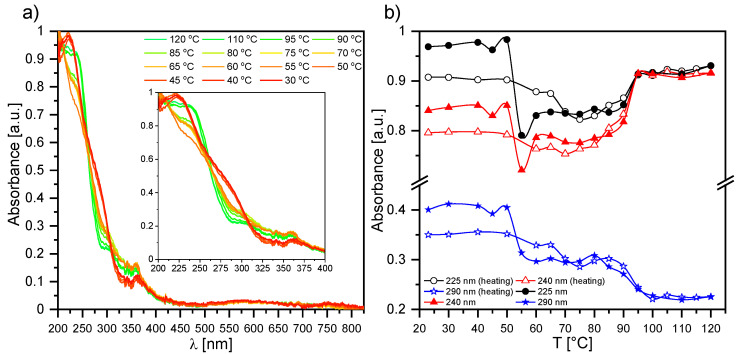
Solid state UV-vis absorption spectra of Composite 1 (pure EHPDB) at several chosen temperatures in the 200–850 nm range during cooling (**a**), and temperature dependence of the absorbance at around 225, 240 and 290 nm (**b**). The inset in graph (**a**) presents an enlarged region of 200–400 nm.

Temperature’s influence on the absorbance of a pure ferroelectric liquid crystal EHPDB (Composite 1) was investigated during heating and subsequent cooling (23 °C–120 °C–23 °C). As is seen in Figure 2a, one absorption band in the UV region with a maximum around 225 nm was well visible for Composite 1 at room temperature. Figure S1 presents the absorption spectra registered for several chosen temperatures during heating of Composite 1, and for better clarity, the temperature dependence of the first derivative of the absorbance for selected wavelengths. After heating above 70 °C (see Figure 2b and Figure S1), for wavelengths of 225 nm, 240 nm, and 290 nm, the absorbance slightly decreased, which was related to the phase transition of crystal to liquid crystalline state, and was in good agreement with the DSC results [39]. Upon further heating, the absorbance increased for wavelengths of 225 nm and 240 nm exponentially up to about 95 °C, above which the absorbance stabilized up to at least 120 °C (Figure 2b). This behavior was related to the phase transition from the liquid crystalline state to an isotropic liquid, and was also in agreement with the DSC results [39]. Interestingly, the absorbance for the wavelength of 290 nm behaved quite differently during further heating; from the phase transition to the SmC* phase, it monotonically decreases. Upon subsequent cooling, the spectra remain unchanged down to 95 °C, while below this value the absorbance for 225 nm and 240 nm decreased with decreasing temperature, down to ca. 50 °C. In turn, for the wavelength of 290 nm, there was an increase in the absorbance up to the crystal phase. A clear jump at the phase transition to crystal phase was well visible in the absorbance for all mentioned wavelengths. As is seen in Figure 2a, the sample became a nonabsorbent material from ca. 425 nm, and only at ca. 575 nm and 750 nm did the absorbance begin to slight increase, but it was still at a level of 5%.

#### 2.2.3. UV-Vis Results for Composites 2–5

**Figure 3:**
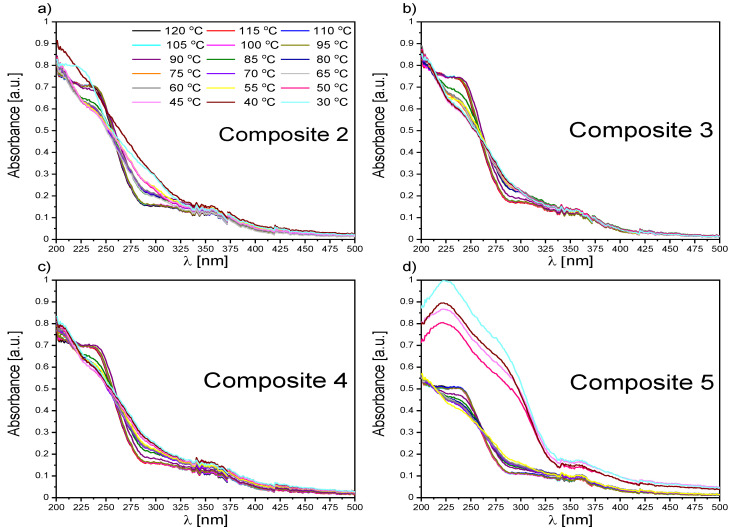
Solid state UV-vis absorption spectra of Composite 2 (**a**), Composite 3 (**b**), Composite 4 (**c**), and Composite 5 (**d**) at several chosen temperatures during cooling. The legend in graph (**a**) is the same for all graphs.

**Figure 4:**
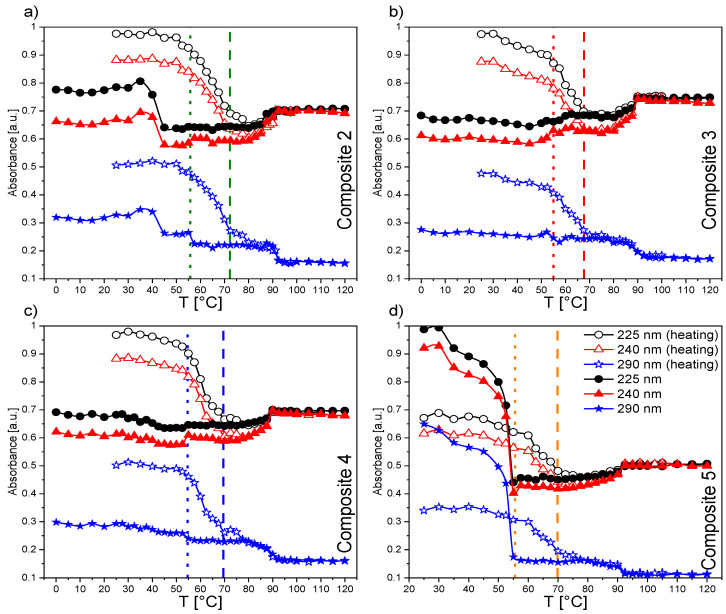
Influence of heating and cooling on the absorbance at 225, 240, and 290 nm for Composite 2 (**a**), Composite 3 (**b**), Composite 4 (**c**), and Composite 5 (**d**). The legend in graph (**d**) is the same for all graphs. The vertical dashed and dotted lines represent the SmA*–SmC* and SmC*–Cr_1_ phase transition temperatures (during cooling), respectively.

The main difference in the spectra of Composites 2–5 (Figure S2) and pure EHPDB—Composite 1 (Figure 2a) was the presence of additional bands (in the range of 350–600 nm) with small intensities corresponding to the absorption of pure nanoparticles (Figure 1a). The temperature evolutions of absorption spectra for Composites 2–5 (see Figure 3 and Figure 4 and Figure S3) were very similar to that of pure EHPDB. The monotonous decrease in absorbance in the visible region upon heating can be explained by the reorientation of the nanoparticles and liquid crystal molecules at the phase transition from solid to liquid crystalline phase. After this phase transition, the absorbance for the wavelengths 225 nm and 240 nm slightly increased and stabilized at the temperature of the phase transition to the isotopic liquid. Such behavior was observed for all nanocomposites. Interestingly, for the 290 nm wavelength, the situation was completely different for all nanocomposites; there was a continuous decrease in the absorbance from the crystal–liquid crystalline phase transition, with a distinct jump at the liquid crystal–isotropic phase transition, and stabilization above this temperature. On the other hand, during cooling just below the isotropic liquid–cholesteric phase transition, the absorbance for the wavelengths 225 nm and 240 nm slightly decreased and reached a plateau throughout the SmC* phase, and at the transition to the crystal phase, a jump was clear visible. However, a decrease in absorbance only for Composite 3, while an almost 50% increase in absorbance for Composite 5, was visible for these wavelengths. In the case of the wavelength at 290 nm, a plateau within the SmC* phase and an increase in the absorbance during crystallization were registered. It is worth noting that the absorbances at 225 nm, 240 nm, and 290 nm were much lower (even by about 30%) for Composite 5 both during heating and cooling as compared to the other nanocomposites (particularly in the isotropic liquid phase and to a smaller extent in the SmC* phase).

Both the undoped sample (Composite 1) and doped samples (Composites 2–5) showed no absorption of incident light in the 500–850 nm range (the absorbance was below 5%; however, this spectral range had a low signal-to-noise ratio, regardless of temperature), which means that Composites 1–5 were transparent in this range. Figure 3 presents the spectral range of 200–500 nm and a high absorbance of the created nanocomposites (Composites 2–5) in the UV region, indicating almost complete absorption of incident radiation. However, a decrease in absorbance with an increase in the *γ*-Fe_2_O_3_ admixture concentration was visible. The nanoparticles in the organic matrix most likely acted as a scattering agent in the UV region. As is seen in Figure S3, the temperatures at which the absorbance changed corresponded to the phase transition temperatures obtained by the DSC method [39].

**Figure 5:**
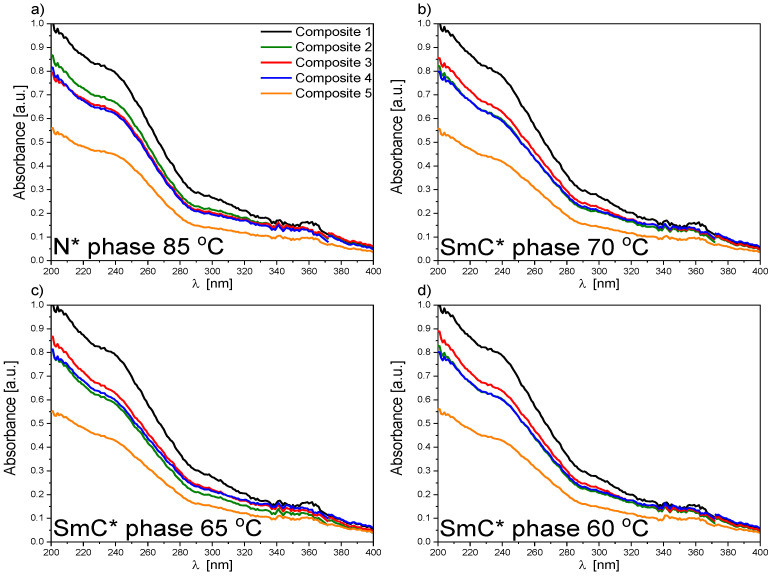
The UV-vis absorption spectra (200–400 nm) for all studied nanocomposites registered during cooling in the N* phase at 85.0 °C (**a**) and the SmC* phase at 70.0 °C (**b**), 65.0 °C (**c**), and 60.0 °C (**d**). The legend in graph (**a**) is the same for all graphs.

Figure 5 presents the spectra recorded during cooling in the N* and SmC* phases for Composites 1–5. In both the N* and SmC* phases, the highest absorbance was observed for Composite 1, and the lowest for Composite 5. Only for Composite 3 was an increase in absorbance in the SmC* phase compared to the N* phase clearly visible, while similar absorbance in these phases was shown for Composites 2 and 4. For Composite 5, a decrease in absorbance by as much as 45% relative to the pure matrix was observed, and this difference for each temperature became smaller for longer wavelengths. To the best of our knowledge, no *γ*-Fe_2_O_3_ nanoparticles showed plasmon resonance, which could explain the changes in absorbance for Composites 2–5 relative to pure EHPDB. Except for Composite 2, there was a well-observed trend: a decrease in the absorbance with an increase in the nanoparticle concentration. Interestingly, the difference in absorbance for Composites 4 and 5 even reached about 20%. Such observations led to the conclusion that the nanoparticles acted as scattering centers for the incident light in the EHPDB matrix. It seemed that single nanoparticles and small aggregates in the matrix reacted differently to the incident radiation. The highest nanoparticle concentration and the presence of aggregates (Composite 5) were responsible for the scattering of the incident light to the highest extent. On the other hand, the qualitative nature of the spectra recorded in the N* phase was very similar to those recorded in the SmC* phase, except for Composite 2.

#### 2.2.4. UV-Vis Results for Composite 6

**Figure 6:**
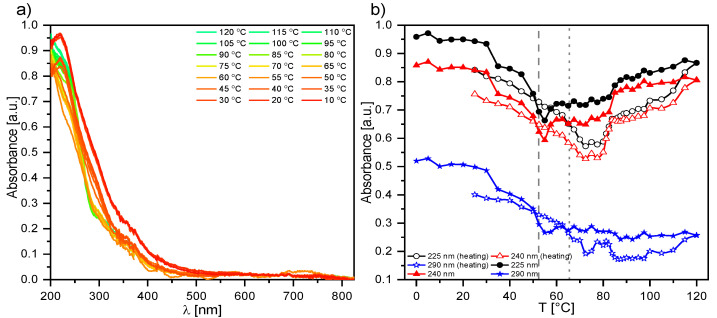
Solid state UV-vis absorption spectra of Composite 6 (*γ*-Fe_2_O_3_, 0.3 wt % + oleic acid) at several chosen temperatures in the 200–850 nm range (**a**), and the influence of heating and cooling on the absorbance at 225, 240, and 290 nm (**b**). The vertical dashed and dotted lines represent the SmA*–SmC* and N*–SmA* phase transition temperatures (during cooling), respectively.

The temperature evolution of the UV-vis absorption spectrum of Composite 6 (Figure 6a) was not significantly different from that of Composites 2–5. The differences that were worth paying attention to were higher absorbance (especially in the UV region during cooling) and lower during heating (Figure S4a) in comparison to Composite 2 (the same concentration of *γ*-Fe_2_O_3_, but without oleic acid). Similarly to other nanocomposites, Composite 6 was transparent from about 400 nm, and no plateau was observed for 225 nm and 240 nm in the isotropic liquid. A slight decrease in the melting point in relation to Composite 2 (Figure S4) was visible.

#### 2.2.5. Theoretical Calculations of UV-Vis and Energy Level Diagram

The structural parameters and energy level diagrams of an organic liquid crystal can be strongly influenced by the electronic nature and position of chemical groups, as well as the relative position of the molecules in the solid state [43]. Geometrical optimalization of an EHPDB molecule was performed in the gas phase using the B3LYP method for the basis set cc-pVDZ embedded in the Gaussian16 software (Figure S5 and Table S1) [44,45]. Consequently, the nonplanar character of this molecule was confirmed, with a dihedral angle of 46° between the aromatic rings of parts ‘B’ and ‘C’ (Figure S5). Moreover, the energy level diagram for EHPDB was determined, resulting in a HOMO (the highest occupied molecular orbital)-LUMO (the lowest unoccupied molecular orbital) gap equal to 4.67192eV, which indicated the very high kinetic stability and low chemical reactivity of EHPDB, as well as a high value of global chemical hardness (*ƞ* = 2.33596 eV) compared to other liquid crystals [43]. Additional time-dependent density functional theory (TD-DFT) calculations were performed for the lowest 100 electronic transitions, yielding the oscillator strength and corresponding molecular orbitals (Table S2). The analysis of these data confirmed that electronic transitions occurred exclusively in the UV region (Figure S5): ~300 nm (123:HOMO→124:LUMO transition involving π-electron transfer from ‘B’ to ‘C’ part of EHPDB), ~255 nm (122:HOMO-1→124:LUMO and 123:HOMO→125:LUMO + 1/126:LUMO + 2 transition involving the transfer of π-electrons within the ‘C’ part, and from the ‘B’ part to the ‘B’ or ‘C’ parts, respectively), ~215 nm (123:HOMO→127:LUMO + 3 and 120:HOMO-3→124:LUMO and transition involving the transfer of π-electrons within the ‘B’ part, and from the ‘B’ to ‘C’ part, respectively), and ~190 nm (121:HOMO-2→126:LUMO + 2 and 120:HOMO-3→125:LUMO + 1 transition of π-electrons from the ‘A’ to ‘C’ part, and within the ‘B’ part). Moreover, the calculated spectrum was shifted to a higher energy by approximately 30 nm compared to the experimental spectra, due to the applied single-point DFT calculation method, which, despite omitting the periodicity of the structure in the liquid crystal phases, still accurately reflected the experimental spectra of Composites 1–6.

In order to determine the possible origin of the shift of the UV-vis absorption spectra of Composites 1–6 towards lower energies after cooling, a similar calculation for the second model (‘planar’ EHPDB molecule) with a dihedral angle close to 0° between the aromatic rings of the ‘B’ and ‘C’ parts was performed (Tables S3 and S4 and Figure S6). This approach showed that the decrease in the dihedral angle did not affect the electronic structure of the frontier molecular orbitals, but significantly decreased the HOMO-LUMO energy gap (4.22293 eV) [46]. Consequently, the calculated UV-vis spectrum for the ‘planar’ EHPDB molecule was shifted to higher wavelengths in the UV region. These results suggested a potential explanation for changes in the UV-vis spectra as a result of the mutual orientation of the aromatic rings within the liquid crystal molecule caused by phase transitions. In the crystalline phase, π-stacking between the aromatic rings and hydrogen bonds was expected to stabilize the planar structure. Next, during heating, thermal motions overcame weak interactions and led to an augment of the dihedral angle and the energy gap values, as well as a shift of the absorption spectrum towards lower energies. Finally, the cooling process led to the reverse behavior.

### 2.3. Frequency Domain Dielectric Spectroscopy (FDDS) Results

The analysis of the dielectric spectroscopy results was limited to the liquid crystalline and crystal phases, as they were the most interesting. Particular emphasis was placed on the temperature range of the ferroelectric SmC* phase to determine how the *γ*-Fe_2_O_3_ nanoparticle admixture affected the relaxation processes present in the LC matrix, and whether they extinguished them or whether new ones appeared. Because the range of the SmC* phase was very narrow, and it was impossible to obtain a uniformly planar orientation for Composite 6, the dielectric properties of this composite were not studied [39].

**Figure 7:**
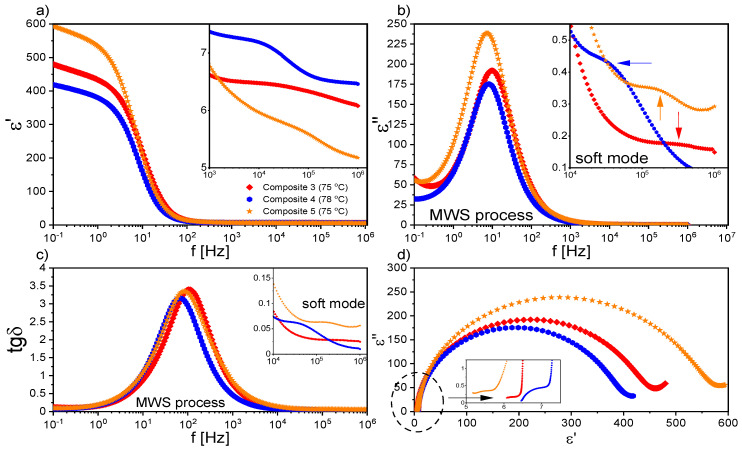
Dielectric dispersion (**a**) and absorption (**b**), loss tangent versus frequency (**c**), and Cole–Cole plot (**d**) for Composites 3–5 in the TGBA*/SmA* phase. Insets present the high-frequency range. The legend in graph (**a**) is the same for all graphs.

**Figure 8:**
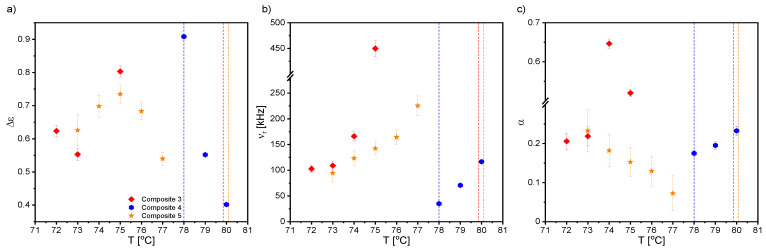
Temperature dependence of the dielectric increment, Δε (**a**); the relaxation frequency, ν_r_ (**b**); and the α distribution parameter of the relaxation time (**c**) of the SM for Composites 3–5. The legend in graph (**a**) is the same for all graphs. The vertical dashed lines represent the N*–TGBA*–SmA* phase transition temperature (during cooling), respectively.

Starting with the first liquid crystalline phase during cooling, two relaxation processes were registered for Composites 2–5 in the N* phase. The mode with a very large dielectric increment in the low frequency range was interpreted as the Maxwell–Wagner–Sillars (MWS) relaxation process; while in the higher frequency range, as the molecular s-process (reorientation around a short molecular axis). However, due to the limited measuring range (up to 10^6^ Hz), the molecular process was partially observed only for Composites 2–5, although we reported in our previous paper on the molecular s-process for pure EHPDB in the range of 10^6^–10^7^ Hz [47]. The relaxation frequency of this molecular process for Composites 2–5 was lower than for Composite 1 (pure matrix), which meant that nanoparticles hindered reorientation around the short molecular axis. In the TGBA* and SmA* phases, apart from the low-frequency MWS process, the soft mode (SM) was registered for Composite 1 and Composites 3–5, while for Composite 2, it was not. Figure 7 presents the dielectric dispersion and absorption, the loss tangent (tgδ = ε’’/ε’), and the Cole–Cole plots for Composites 3–5 in the TGBA*/SmA* phase. It was seen that the MWS process was dominant in the low-frequency range; while at higher frequencies, the soft mode was visible (in the insets). As is known, the relaxation frequency for this process is temperature-dependent, especially in the vicinity of the SmA*–SmC* phase transition, where the V-shaped behavior is usually observed [48,49,50]. For Composites 3–5, the full V-shape behavior of the relaxation frequency was not visible (see Figure 8b), as the SM disappeared completely after phase transition to the SmC* phase.

Temperature dependence of the dielectric increment, Δε; the relaxation frequency, ν_r_; and the distribution parameter of the relaxation time, α, obtained by fitting the Cole–Cole model [51] to the registered dielectric spectra in the TGBA*/SmA* phases are presented in Figure 8. The α parameter for SM was mostly in the range of 0.1–0.2, and only for Composite 3 was above 0.5 (Figure 7c). The small dielectric increment and strongly temperature-dependent relaxation frequency were characteristic of the soft mode, and was reported in the literature, e.g., for ferroelectric liquid crystal 10.OPOSMH [52].

**Figure 9:**
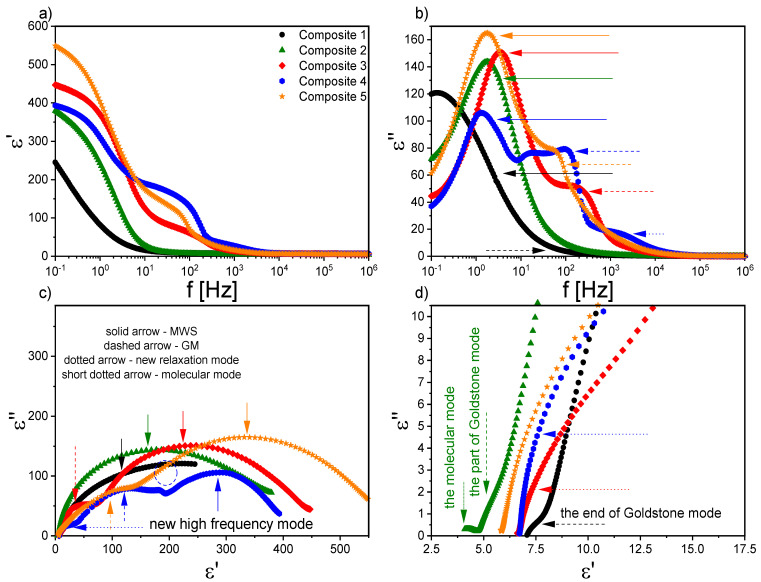
Dielectric spectra of Composites 1–5 in the ferroelectric SmC* phase (60 °C): dispersion (**a**); absorption (**b**); Cole–Cole plot (**c**); enlarged high-frequency part of Cole–Cole plot (**d**). Continuous colored arrows indicate the MWS process, dashed—the GM, dotted—a new relaxation process, and short-dotted—a molecular process. The legend in graph (**a**) is the same for all graphs. The dashed blue circle in graph (**c**) indicates an additional relaxation process present between the MWS and the Goldstone processes for Composite 4.

Figure 9a,b present the dielectric spectra registered in the ferroelectric SmC* phase for Composites 1–5, while Cole–Cole plots are presented in Figure 9c,d. The dominant MWS process in the low-frequency range was well visible. In the SmC* phase, in addition to the MWS process, the Goldstone mode (GM) was registered in the nanocomposites (except for Composite 2), which was related to the phase fluctuation of the director in the smectic layer [53]. It seemed that the GM was strongly suppressed and covered by the MWS process in Composite 2 (see Figure 9d); therefore, the determination of its dielectric parameters was not possible. For Composite 1, up to 60 °C, the MWS and GM processes were observed. At lower temperatures, the MWS relaxation frequency strongly depended on temperature, and shifted to lower frequencies (while simultaneously, the dielectric increment decreased). For Composite 2 in the SmC* phase, the MWS process was observed, and a very weak dielectric process (most likely a molecular one) in a higher frequency range. In turn, for Composites 4–5, apart from the MWS and the GM processes, a weak dielectric process (most likely a molecular one) in a higher frequency range was registered in the SmC* phase (marked as new high-frequency mode in Figure 9c). In the case of Composite 4, in the vicinity of 63 °C, another relaxation process was revealed between the MWS and the GM processes (surrounded by the dashed circle in Figure 9c). As is seen in Figure 9a, the dielectric constant increased significantly after doping, while Khushboo et al. observed its decrease by more than half in the ferroelectric liquid crystal mixture W206E doped with Fe nanoparticles (0.1 wt %) [54].

The influence of nanodopants in the liquid crystal matrix on relaxation processes is reported in the literature; for example, Singh et al., by doping the ferroelectric liquid crystalline mixture Felix 16/100 with quantum dots of CdSe (ca. 3.5 nm, 1 and 2 wt %), registered a new temperature-dependent relaxation process for a nanocomposite with 1 wt % CdSe, but for 2 wt % CdSe, did not. This process was explained by quantum fluctuations, which were suppressed at higher concentrations. In turn, after doping, the GM process became broader, and shifted towards lower frequencies, while the dielectric increment and the specific electrical conductivity decreased, which was explained by the trapping of ion impurities by QDs with strong adsorption abilities [55]. On the other hand, the nanocomposites based on Felix 17/100 doped with CdSe (ca. 3.5 nm, 1 and 2 wt %) showed a decrease in P_s_, W_D_, and W_P_; an increase in rotational viscosity; homeotropic alignment of FLC molecules; and a new ionic relaxation process around 300 Hz, which was interpreted as the adsorption phenomenon or accumulation of ion carriers on the QDs’ surface [56]. In our case, for Composite 4, the relaxation process between MWS and GM at a frequency around 20 Hz (60 °C) may have a similar origin, e.g., accumulation of ion carriers on the *γ*-Fe_2_O_3_ nanoparticles’ surfaces. It cannot be ruled out that this process was related to certain movements of nanoparticle aggregates or the joint movement of LC molecules with the surrounding nanoparticles (or their aggregates). In turn, the relaxation frequency of the new high-frequency mode in Composite 4 (Figure 9c) was higher than that of GM, as it was observed for the relaxation process in Felix 17/100 doped with a higher concentration of ZnS QDs [38]. This relaxation process was attributed to the flexoelectric tilt fluctuations in the host in the vicinity of QDs, and it may be possible that for Composite 4, the origin of the new high-frequency relaxation mode was the same.

**Figure 10:**
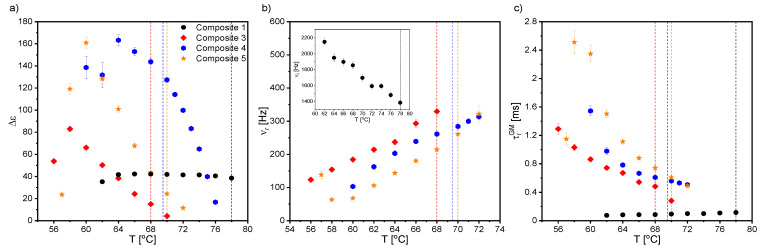
Temperature dependencies of the dielectric increment, Δε (**a**), relaxation time (**b**) and relaxation time (**c**) of the Goldstone mode in nanocomposites. The legend in graph (**a**) is the same for all graphs. The vertical dashed lines represent the SmA*–SmC* phase transition temperature (during cooling).

The temperature dependencies of the dielectric increment and relaxation times of the GM for Composites 1 and 3–5 are presented in Figure 10. The dielectric increment, as well as the relaxation time for Composite 1, was temperature independent, which was characteristic of the Goldstone mode. Moreover, the relaxation time was the same order as that of another pure ferroelectric liquid crystal, 3FO6C1 [57]. In contrast, the relaxation frequency of the Goldstone mode in nanocomposites was at the level of a pure C8 compound over a wide temperature range [58]. Different behaviors in the temperature dependence of both the dielectric increment and relaxation times took place for Composites 3–5—they were strongly temperature-dependent, and higher than for Composite 1. The dielectric increment after admixture increased 4-fold (up to ca. 180); however, there is a known GM with an even higher Δε in pure material [59]. Importantly, as the nanoparticle concentration increased, the relaxation time for the GM also increased. Extending the GM relaxation time in Composites 3–5 compared to Composite 1 was associated with difficulties in movement at the molecular level after doping with nanoparticles. The higher the concentration of nanoparticles in the matrix, the more difficult or inhibited the movement of LC molecules in the vicinity of the nanoparticles was. Ibragimov et al. doped 5CB and H37 liquid crystals with BaTiO_3_ particles (ca. 600 nm) and also observed the increase in the relaxation times after doping [60]. This was explained by a strong interaction existing between organic molecules and the BaTiO_3_ particles (most likely permanent dipole—permanent dipole). Due to interacting organic molecules and dispersed particles, the rotation of molecules was hindered in the vicinity of the particles. As a result, the frequency was shifted to lower values. A similar explanation for the longer relaxation time of the GM after doping with *γ*-Fe_2_O_3_ nanoparticles can be adapted in our case; however, the interaction’s nature could be different (e.g., permanent dipole—induced dipole). A similar behavior of the GM’s parameters was observed in pure compounds from the homologous series 3FnHBM6(S), as well as in pure K1, K2, and K3 compounds [61,62]. In turn, Gathania et al. doped the ferroelectric mixture FLC-6980 with a dichroic dye (2wt %) and obtained ca. 3- and 5-fold increases in the SM and GM dielectric increments, respectively [63]. It is also known that the relaxation time is directly proportional to the rotational viscosity, *γ,* and helix pitch, *p,* while inversely proportional to the elastic constant, *K_3_*. Thus, the observed change in the GM relaxation time with the temperature for Composites 3–5 may have resulted from the competition between the temperature dependence of *K_3_, p,* and *γ* in the SmC* phase. The temperature dependencies of *p* and *γ* for different admixture concentrations were determined by us (see our last article [39]); however, due to the lack of *K_3_* temperature dependence, we were not able to confirm this estimate.

It should also be noted here that the existence of a domain mode (DM) in Composites 2–5 could not be ruled out, as we observed striped textures of the SmC* phase for these composites [39]. Haase et al. reported on the possibility of observing ferroelectric domains under a polarizing microscope in the form of a striped texture [64], while DM appeared for an already partially unwounded helix under an external electric field, and was associated with the formation of ferroelectric domains (dipole moments from several adjacent smectic layers were directed in one direction) [65].

**Figure 11:**
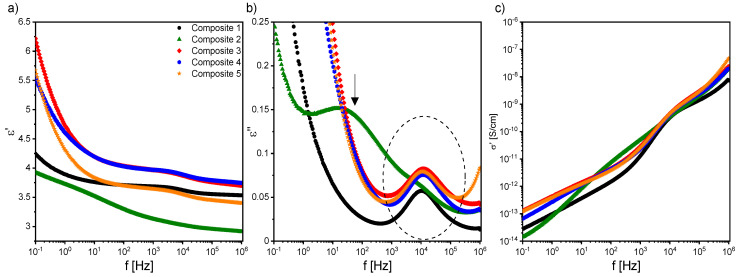
Dielectric dispersion (**a**) and absorption (**b**), as well as real component of the specific electric conductivity (**c**) for Composites 1–5 in the crystal phase (30 °C). The vertical arrow in (**b**) indicates the additional process registered for Composite 2. The legend in graph (**a**) is the same for all graphs. The dashed ellipse in (**b**) surrounds the high-frequency process present for all nanocomposites in the crystal phase.

During further cooling, in the vicinity of the transition to the crystal phase, the MWS process disappeared for all nanocomposites, while another relaxation process (at ca. 10^4^ Hz) was registered in the crystal phase down to 25 °C. Figure 11 presents the dielectric spectra and the real component of the specific electric conductivity registered for all nanocomposites in the crystal phase. As is seen in Figure 11b, the relaxation frequency of this process weakly depended on the nanoparticles concentration, while its intensity increased after doping. This process could not be attributed to the presence of nanoparticles in the LC matrix, as it was also observed for Composite 1 (pure matrix). Its relatively high relaxation frequency instead suggested that it may have been associated with the impurities (not ions) existing in the EHPDB (purity ca. 98%, according to the manufacturer).

An additional strong relaxation process, strongly temperature-dependent, was registered only for Composite 2 in the crystal phase (indicated by the arrow in Figure 11b). This process covered the high-frequency process (see the dashed ellipse in Figure 11b) present for all nanocomposites in the crystal phase. Its relaxation frequency decreased with decreasing temperature by almost three orders of magnitude. The nature of this process is still unknown. Similarly, the additional relaxation process was observed by Goel et al. after doping an LC matrix with Ni nanoparticles, and was explained by exchange interactions for higher nanoparticle concentrations [66]. The specific electric conductivity in the crystal phase changes was quite irregular with frequency for Composites 1–5 (Figure 11c); however, an admixture effect on its increase was visible (except at a few points at the low-frequency limit for Composite 2, which could have been due to the existence of the strong low-frequency process in this nanocomposite).

**Figure 12:**
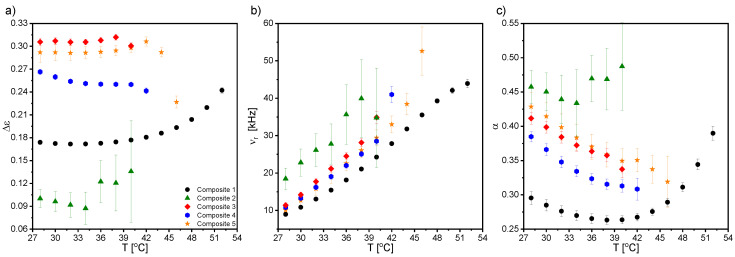
Temperature dependences of the dielectric increment, Δε (**a**); relaxation frequency, ν_r_ (**b**); and distribution parameter of relaxation time, α (**c**) of the relaxation process registered in the crystal phase. Due to the overlapping of two processes for Composite 2, the parameters were subject to large uncertainties. The legend in graph (**a**) is the same for all graphs.

Figure 12 presents temperature dependencies of the Δε, ν_r_, and α parameters of the high-frequency relaxation process observed in the crystal phase for all nanocomposites (in the case of Composite 2, the double Cole–Cole function was fitted). As is seen, this relaxation process was visible at cooling just from the SmC*–Cr phase transition for Composite 1, while it appeared a few degrees below this transition for Composites 2–5. Its dielectric parameters (Δε, ν_r_, and α) increased after doping the LC matrix with *γ*-Fe_2_O_3_ nanoparticles, except for Composite 2 (due to the overlapping of the low- and high-frequency processes, they were subjected to large uncertainties). The strong modifications of the α parameter were due to the distortion of the long-range order in the crystal phase.

As mentioned above, a very strong Maxwell–Wagner–Sillars (MWS) process was registered in the low-frequency range in the isotropic liquid, as well as in all liquid crystalline phases. Charge carriers in the samples (e.g., ionic impurities) could be blocked on the internal dielectric boundary layers on the mesoscopic scale, which was manifested in the dielectric spectrum as the Maxwell–Wagner–Sillars process [67]. The MWS process must be considered, especially in heterogeneous materials such as colloids, biological materials, blends, liquid crystal polymers, and composite systems [68]. In addition, charge carriers in contact with the nanocomposites can accumulate on the outer electrodes, which in turn is manifested by a strong increase in, among others, dielectric dispersion, and is called electrode polarization (EP, on a macroscopic scale) [67]. In the case of MWS and EP, the charge carriers were separated and made separate contributions to the polarization. In both cases, the contribution to the dielectric losses was high. It is also well known that ions present in organic materials modify the various physical properties, including the conductivity. After applying an electric field to the sample, mobile ions (coming from LC matrix or nanoparticles) moved to the electrodes of the measuring cell, and the local field acting on LC molecules was smaller than applied field. Moreover, this ion separation (electrode polarization) caused a continuous increase in dielectric dispersion, ε’, which could reach enormous values, especially in the low-frequency range. However, in Composites 1–5, in the low-frequency range the EP contribution was quite small, and it could be seen as a nonplateau region (see Figure 7 and Figure 9). A contribution to the MWS process also was made by the polarization relaxation taking place at the interface between two various materials with different dielectric constants. A characteristic feature of the MWS process is the increase in the dielectric dispersion in the low-frequency range.

**Figure 13:**
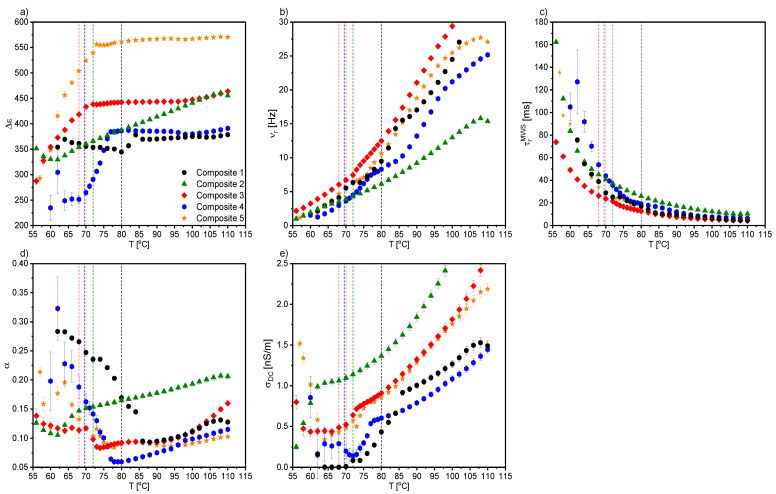
Temperature dependencies of the dielectric increment, Δε (**a**); relaxation frequency, ν_r_ (**b**); relaxation time (**c**); the distribution parameter of relaxation time, α (**d**) of the Maxwell–Wagner–Sillars process; as well as DC conductivity (**e**) for Composites 1–5. The legend in graph (**a**) is the same for all graphs. The vertical dashed lines represent the SmA*–SmC* phase transition temperature (during cooling).

As mentioned above, the MWS relaxation process originated from the relaxation of the polarization at the interface between different dielectric materials [67]. In the case of created nanocomposites, this interface was between (1) liquid crystal and *γ*-Fe_2_O_3_ nanoparticles and (2) liquid crystal and polymer alignment layer on the sample electrodes. After applying the electric field to the measuring cell, the surface charges accumulated at these interfaces, and the Debye-type MWS relaxation process could be registered [67]. In turn, the interface between the liquid crystal and electrode was related to the electrode polarization phenomenon at the low-frequency limit. Interfaces acted as capacitors (with small plate distances), so finally the formation of a super capacitor at low frequencies (ca. 1Hz) and hence rapid increase in the dielectric dispersion (parasitic effect) were observed. Usually, two or more relaxation processes and conductivity contributions are observed in the dielectric spectrum. If the conductivity is of electronic origin, it does not influence the dielectric dispersion, ε’ [67], but does affect the dielectric absorption, ε’’ (ε’’ = σ_0_/(2πfε_0_); σ_0_ is the DC conductivity). In the case of ionic charge carriers, the dielectric absorption satisfied the relationship ε’’~ν^−s^ (s < 1, ν is frequency). These ionic charge carriers were responsible for electrode polarization or Maxwell–Wagner–Sillars relaxation. For the electronic conductivity contribution (ohmic conductivity), the slope of ε’’ in the low-frequency range was equal to −1, while in the case of nonohmic conductivity or polarization effects (MWS and/or EP), the slope was greater than −1 (at higher frequencies, the conductivity contribution slope is equal to −1) [67]. Of course, the electrode polarization was also associated with an increase in the dielectric dispersion. Taking all of the above into account, the Cole-Cole model with the DC conductivity contribution was fitted to the experimental data, and the results obtained for the MWS process are presented in Figure 13. As is seen, the dielectric increment was temperature independent in the high-temperature range for all Composites, except for Composite 2. The change in the temperature dependence of Δε, at different temperatures, for Composites 1 and 3–5 was related to the SmA*–SmC* phase transition, while a monotonous decrease was observed for Composite 2.

Additionally, both the dielectric increment and ε_s_ were higher for nanocomposites than for pure EHPDB (Composite 1), which was due to the interfacial polarization (at the liquid crystal–nanoparticles interface). However, Katiyar et al. obtained a decrease in relative permittivity while doping nematic liquid crystal NLC 2020 with TiO_2_ nanoparticles (ca. 20–24 nm, anatase), which was explained by the ion trapping phenomenon by nanoparticles [69]. The relaxation frequency of the MWS process increased with increasing temperature for all composites, and the highest was for Composite 3. Such behavior was also observed earlier [70]. As mentioned above, the MWS process was a Debye type, therefore the α parameter should be equal to 0, and the Cole–Cole plot should be in the form of a semicircle with the center on the ε’ axis [71]. For Composites 1 and 3–5, the α parameter was small (especially at 75–100 °C); while for Composite 2, it was much higher (due to, inter alia, the overlapping of the MWS and Goldstone processes). Goswami et al,. as well as Czerwiec et al., showed that for the ferroelectric liquid crystals 6F3R and 6F6BbiOC8, the relaxation frequency of the MWS process was very weakly dependent on temperature, and the dielectric increment was greater than for the Goldstone process [72,73]. In Figure 13e, it is well visible that the DC conductivity decreased with decreasing temperature. Within the measurement uncertainty, the electronic conductivity was the same for Composites 3 and 5. However, for Composite 4 up to 82 °C was the smallest. Throughout the temperature range of the SmC* phase, the DC conductivity was higher for Composites 2–5 compared to the pure matrix (Composite 1).

**Figure 14:**
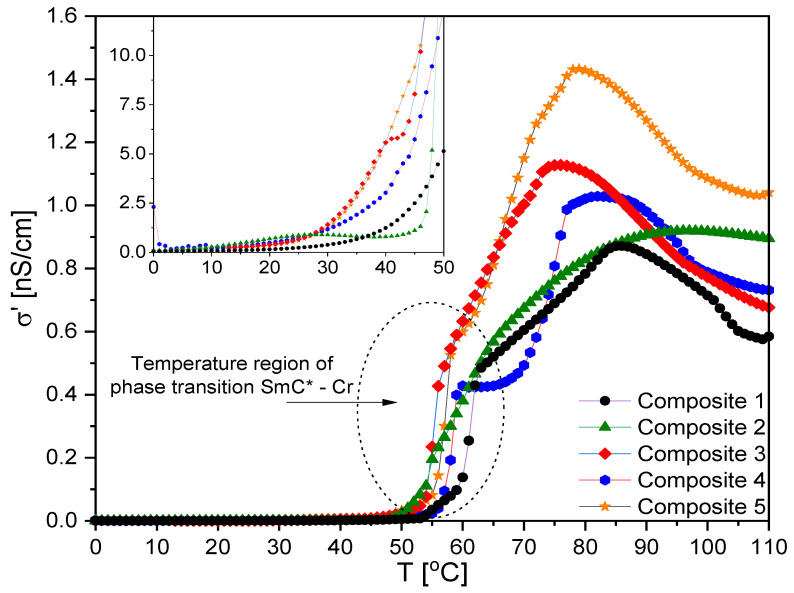
Temperature dependencies of the real part of specific electric conductivity for Composites 1–5 registered at 10.5 Hz.

Another interesting issue from the application point of view was the specific electric conductivity, σ*. As is seen in Figure 14, the real part of σ* (σ* = σ’ − *i*σ” [74]) for nanocomposites was higher than for the pure matrix. It should also be kept in mind that the conductivity could also be affected by ionic impurities adsorbed on nanoparticles and introduced into the matrix at the synthesis stage. At higher temperatures, they desorbed from the nanoparticles’ surfaces and become parasitic ions in the matrix [75]. It is very interesting that σ’ was much higher in mesophases than in the crystal phase, and the differences were enormous. For example, for Composite 5, the difference in σ’ between the crystal and liquid crystalline phases was around 1500 pS/cm. Within mesophases, the highest specific electric conductivity was for Composite 5, as was expected for the nanocomposite with the highest amount of *γ*-Fe_2_O_3_ nanoparticles. Kumar et al. also observed an increase in conductivity after doping the ferroelectric liquid crystals 5F6T and 6F6T with TiO_2_ nanoparticles (ca. 18–23 nm, 0.1 and 1.0 wt %) [76], as did Naser, after doping liquid crystal with Fe_3_O_4_ nanoparticles (up to 1 wt %) [77].

### 2.4. Superconducting Quantum Interference Device (SQUID) Magnetometry Results

It is known that liquid crystal molecules are usually diamagnets, and in a sufficiently strong magnetic field (3–4 T), they align in the direction of the field forces. The introduction of non-diamagnetic inorganic nanoparticles decreased the magnetic field needed for the reorientation of LC molecules [78]. However, the aim of our study was to find out how the properties of nanoparticles would change after they were introduced into an LC matrix.

Measurements of magnetic properties were used to check whether the synthesized nanocomposites (with small *γ*-Fe_2_O_3_ nanoparticle concentration) exhibited a hysteresis loop present for pure nanoparticles in a wide temperature range (in a blocked state), and how the concentration of *γ*-Fe_2_O_3_ nanoparticles affected the characteristic parameters of the hysteresis loop, such as: saturation magnetization, M_S_; residual magnetization in the zero magnetic field, M_R_; and the coercive field, H_c_. Additionally, we wanted to determine how an admixture modified the magnetization in the ferroelectric SmC* phase of the studied nanocomposites.

Because *γ*-Fe_2_O_3_ nanoparticles were studied, the blocking temperature, T_B_ (phase transition temperature between superparamagnetic and blocked state), existed. In the blocked state (below T_B_), the magnetic moments of the nanoparticles were frozen, and the value of thermal energy was not sufficient to reorient them. Below a certain critical size of *γ*-Fe_2_O_3_ nanoparticles, their magnetic properties changed from ferromagnetic to superparamagnetic (size effect). However, the critical size of nanoparticles depended, inter alia, on the conditions of synthesis. In the superparamagnetic state, the magnetization curve was not a hysteresis loop, magnetization saturation was not observed (even in a high magnetic field), and irreversibility below T_B_ occurred [79].

**Figure 15:**
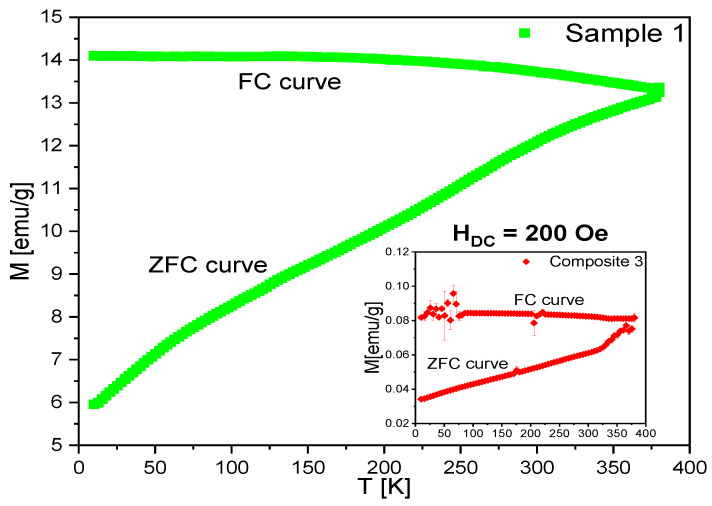
The qualitative temperature dependence of magnetization in the external magnetic field (200 Oe) for Sample 1 (pure *γ*-Fe_2_O_3_ nanoparticles) and Composite 3 (in the inset).

The magnetic properties of Samples 1–2 and Composites 2–6 were investigated by measuring the temperature dependence of magnetization in a DC magnetic field of 200 Oe (ZFC and FC curves; the external magnetic field dependence of magnetization and the magnetic hysteresis (at 10, 100, 200, and 300 K)). As an example, ZFC and FC curves for Sample 1 and Composite 3 are presented in Figure 15. The fluctuations visible in the FC curve for the Composite 3 were probably caused by an excessive temperature rate. Both the ZFC and FC curves for Sample 1 were similar to those of Composite 3 and other nanocomposites, respectively. As is seen, the FC curve reached saturation in a low-temperature range for both samples, which was previously observed for smaller *γ*-Fe_2_O_3_ nanoparticles [79] and was associated with dipolar interactions. Moreover, the convergence of the ZFC and FC curves was not visible up to 380 K; therefore, both Sample 1 and the nanocomposites were in the blocked state (T_B_ corresponded to the convergence point of both the ZFC and FC curves). It seemed that T_B_ was outside of the measuring range, because we did not use any surfactant to decorate the *γ*-Fe_2_O_3_ nanoparticles, in opposition to Kataby et al., who observed significant T_B_ modifications depending on the surfactant used (e.g., stearic acid shifted T_B_ to 280 K in relation to sulfonic acids, where T_B_ was around 120 K) [79], as well as Tanwar et al., who showed that decoration of nanoparticles with oleic acid changed T_B_ (from 95 K up to 15 K in the 0.5 T magnetic field) [80]. It should also be noted that in the case of surfactant-functionalized nanoparticles, the T_B_ depends on the strength of the magnetic field. Moreover, the T_B_ for the same type of nanoparticles may differ depending on the size of the nanoparticles, their decoration or not with a surfactant, as well as possible impurities. Wu et al. showed that the T_B_ for *γ*-Fe_2_O_3_ short nanotubes very strongly depended on the DC magnetic field used to register the ZFC/FC curves (T_B_ decreased from 275 K to 40 K when the applied magnetic field changed from 500 Oe to 5000 Oe) [81]. Summarizing the above, apart from the modification of the phase transition temperatures and the degradation of mesophases [39], this was another reason not to use any surfactant in the preparation of composites.

**Figure 16:**
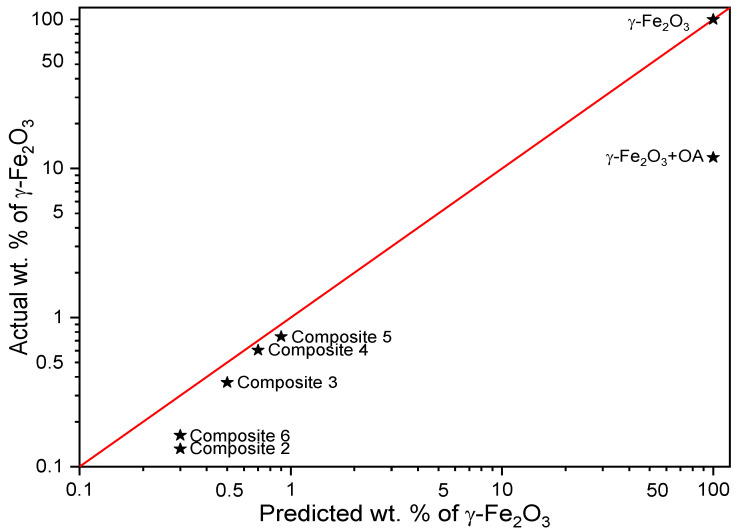
Predicted vs. estimated content of *γ*-Fe_2_O_3_ nanoparticles in the Samples 1–2 and Composites 2–6.

Since all samples consisted of the same *γ*-Fe_2_O_3_ nanoparticles (confirmed by XRD measurements [39]), all experimental data were scaled to the average value of magnetic properties from three independent measurements for pure *γ*-Fe_2_O_3_ nanoparticles. This approach allowed the determination of the estimated weight concentration of *γ*-Fe_2_O_3_ nanoparticles used for the preparation of mixtures with EHPDB and/or oleic acid, as well as a direct comparison of the magnetic properties. The predicted and estimated *γ*-Fe_2_O_3_ nanoparticle content in Samples 1–2 and Composites 2–6 are gathered in Table 1 and plotted in Figure 16. Analysis of these data led to the observation that the estimated concentrations of *γ*-Fe_2_O_3_ nanoparticles in Composites 2–6 were slightly lower than predicted. Such a deviation was a consequence of the partial miscibility of the low-concentrated nanoparticles in the liquid crystal matrix, which led to the deposition of a small amount of *γ*-Fe_2_O_3_ nanoparticles on the glassware surface during the evaporation of the mother solution in chloroform, which, in the last stage of a mechanical collection of the product, resulted in a reduction in the *γ*-Fe_2_O_3_ nanoparticle content. One of the most probable reasons was the formation of nanoparticle aggregates that could no longer be considered as single domains with one super spin, as well as different sizes of nanoparticles in the composites (<50 nm). As mentioned above, the same behavior of ZFC/FC curves was observed for all the samples, so even a small concentration of *γ*-Fe_2_O_3_ in the liquid crystal matrix retained its properties and made the entire nanocomposite form a blocked state up to 380 K. Therefore, it was possible to obtain both a ferroelectric material (because EHPDB exhibited the liquid crystalline SmC* ferroelectric phase) and a magnetically blocked material. In turn, Novotná et al. obtained a ferroelectric–superparamagnetic material with a T_B_ equal to ca. 15 K [82].

It is well known that maghemite (*γ*-Fe_2_O_3_) in bulk exhibits long-range ferromagnetic order with a Curie temperature of about 890 K, a magnetic hysteresis with a coercive field below 200 Oe at room temperature, and saturation magnetization up to 75 emu/g (while lower for *γ*-Fe_2_O_3_ nanoparticles) [81,83,84,85,86,87,88]. Balaraju et al., did not observe the saturation of magnetization of α-Fe_2_O_3_ nanoparticles (ca. 39 nm, rhombohedral structure) up to ±10 kOe [89]. Interestingly, Suber et al., reported on the antiferromagnetic behavior of Fe_2_O_3_ nanoparticles of various sizes and shapes [90]. For example, the Morin phase transition (transition from pure antiferromagnet to ferromagnet) for rhombohedral nanoparticles was observed around 190 K, and then the blocking process at around 60 K; while for spherical nanoparticles, the Morin transition disappeared, and superparamagnetic behavior was observed [90].

**Figure 17:**
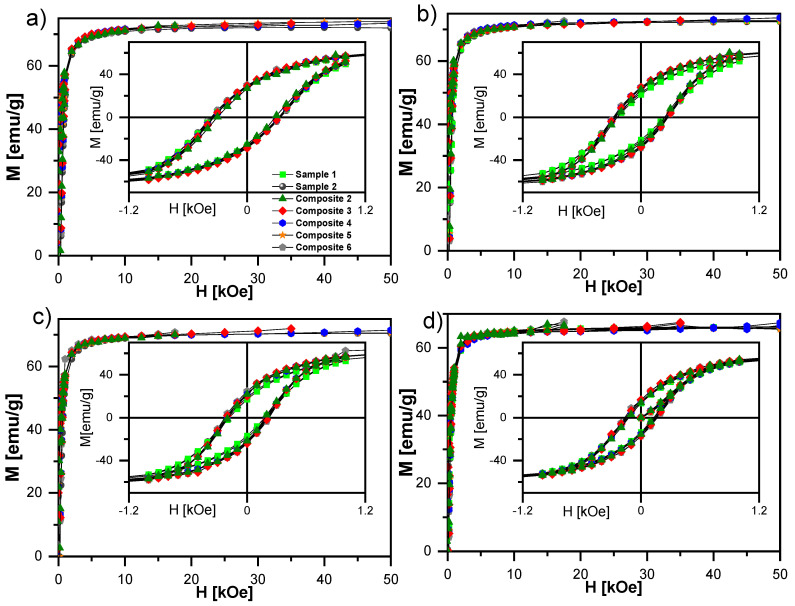
Magnetization vs. external magnetic field and magnetic hysteresis (insets) for Samples 1–2 and Composites 2–6 measured at 10 K (**a**), 100 K (**b**), 200 K (**c**), and 300 K (**d**). All values were scaled to values for the *γ*-Fe_2_O_3_ nanoparticles (Sample 1). The solid lines in all figures are a guide for the eye. The legend in graph (**a**) is the same for all graphs.

The synthesized nanocomposites were indeed hybrid systems, and M(H) curves were measured at various temperatures to confirm the presence of magnetic hysteresis (Figure 17). All compounds revealed a similar shape of magnetic hysteresis in a blocked state (T_B_ > 380 K), with average H_c_ ranging from 129 Oe (Composite 2) to 166 Oe (Composite 6) at 300 K, and successively increasing as the temperature decreased, to reach values between 295 Oe (Composite 2) and 352 Oe (Sample 2) at 10 K. Moreover, the hysteresis loop for each sample narrowed with increasing temperature. Magnetic properties of Samples 1–2 and Composites 2–6 are gathered in Table 2. Both the magnetic remanence, M_R_, and the coercive fields, H_c_, were temperature-dependent. In our case, saturation of magnetization was not observed, even for the highest magnetic field (around 50 kOe). The increase in temperature from 10 K to 300 K caused the magnetization in the highest magnetic field (50 kOe) to decrease from about 75 emu/g to about 65 emu/g. This phenomenon could be explained by the increase in thermal energy that was supplied to the nanocomposite system. The higher thermal energy was responsible for the larger fluctuations at the individual nanoparticle level. This, in turn, means that for a given magnetic field, fewer spins will be oriented towards the external magnetic field, so the magnetization will be lower; e.g., nanoparticles with higher thermal energies have greater freedom of movement, which makes it difficult to organize their spins using the same magnetic field.

**Figure 18:**
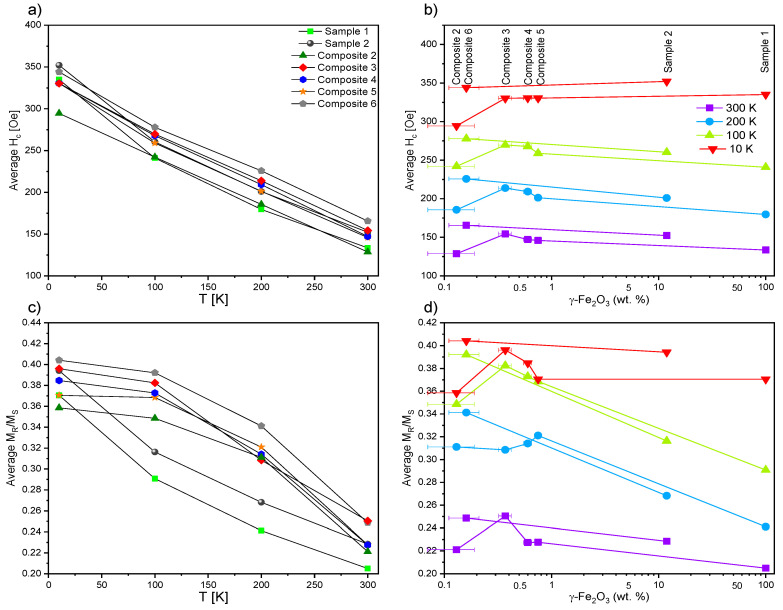
Temperature (**a**) and estimated weight percent (wt %) of the *γ*-Fe_2_O_3_ nanoparticles (**b**) dependencies of the average coercive field, and temperature (**c**) and estimated *γ*-Fe_2_O_3_ wt % (**d**) dependencies of the average remanence ratio (remanent magnetization M_R_/saturation magnetization M_S_) for Samples 1–2 and Composites 2–6. The solid lines in all figures are a guide for the eye. The legend in graph (**a**) is the same for graph (**c**), while the legend in graph (**b**) is the same for graph (**d**).

Moreover, the analysis of the dependence of the average H_c_ in relation to the estimated-actual content of the *γ*-Fe_2_O_3_ nanoparticles (Figure 18b) resulted in the following observations:

(i) An increase in H_c_ for the samples with content ranging between ca. 0.3 wt % and 0.9 wt % (ca. +15% increase in H_c_ for 0.37(5) wt % (Composite 3) in comparison to Sample 1);
(ii) A significant decrease in H_c_ (down to −10%) for the nanocomposite with the lowest concentration of 0.13(6) wt % (Composite 2).

Novotná et al. observed quite different behavior for hysteresis loops, namely narrowing the hysteresis loops for nanocomposites in relation to pure nanoparticles [82]. Of course, in the first approximation, the differences could be explained by different nanoparticle sizes (ca. 4 nm by Novotná et al., and ca. 50 nm in our case).

It is also worth mentioning that oleic acid significantly improved the performance of magnetic hysteresis for materials with decorated nanoparticles, leading to an approximately 12% increase for Sample 2, and approximately 36% for Composite 6. Similar trends with minor deviations were also observed for the average remanence ratio (remanent magnetization M_R_/saturation magnetization M_s_) versus temperature and estimated concentration of *γ*-Fe_2_O_3_ (Figure 18c,d). At each studied temperature, the H_c_ increased while M_R_ and M_S_ decreased for the *γ*-Fe_2_O_3_ sample treated with oleic acid versus the untreated one. In the case of nanocomposites, the H_c_ for the temperatures of 300 K, 200 K, and 100 K was the highest for Composite 3, while no significant differences were observed for the studied samples at a temperature of 10 K. At the lowest temperature saturation of magnetization, M_s_ was the highest for the composite with the highest concentration of *γ*-Fe_2_O_3_ nanoparticles, and the M_s_ for each sample increased with decreasing temperature (which was to be expected). The magnetic properties of the oleic-acid-treated and original samples differed significantly, which is still not well understood by us, but is very exciting for future research using other matrices or other forms of iron (III) oxides (e.g., ε-Fe_2_O_3_). Furthermore, Martínez-Miranda et al. used Fe_48_Co_52_ nanoparticles (ca. 11 nm) decorated with various surfactants as dopants to 8CB, and showed that PEG (3000) (polyethylene glycol) and NHS/PEG (3000) (N-Hydroxysuccinimide/polyethylene glycol) surfactant had different effects on the orientation of 8CB molecules in the magnetic field [78]. Summing up, the magnetic behavior of nanocomposites (Composite 2–5) was similar to that of pure *γ*-Fe_2_O_3_ nanoparticles. Therefore, the nanoparticles’ incorporation into the organic liquid crystalline matrix (EHPDB) did not affect their properties in the blocked state, which was especially visible in the hysteresis loops (Figure 17).

### 2.5. Fourier Transform Mid-Infrared (FT-MIR) Spectroscopy Results

Low amounts of the nanoparticles used in the experiments did not result in sufficiently large structural changes in the liquid crystalline system to be observable by IR spectra at room temperature [39]. However, the nanoparticles, even at such low concentrations, notably affected the thermal response of the system, as revealed by DSC. The changes in the thermal response could be also checked by IR spectroscopy. It is known that IR spectra are extremely sensitive to variations in intermolecular interactions during phase transitions. Thus, IR investigation of phase transitions can bring additional valuable information on the overall supramolecular arrangement of the liquid crystalline system, and suggest possible structural implications of the nanoparticles in intermolecular interactions within the system. Therefore, we investigated IR spectra from 100 °C down to 10 °C upon cooling the system from the isotropic to ordered crystal phase. The purpose of the IR investigations was to draw conclusions about the molecular behavior of the investigated materials by analyzing spectral band parameters such as peak maximum position and full width at half maximum (FWHM) of some IR bands arising from the vibrational modes of various functional groups present in EHPDB in the function of temperature.

**Figure 19:**
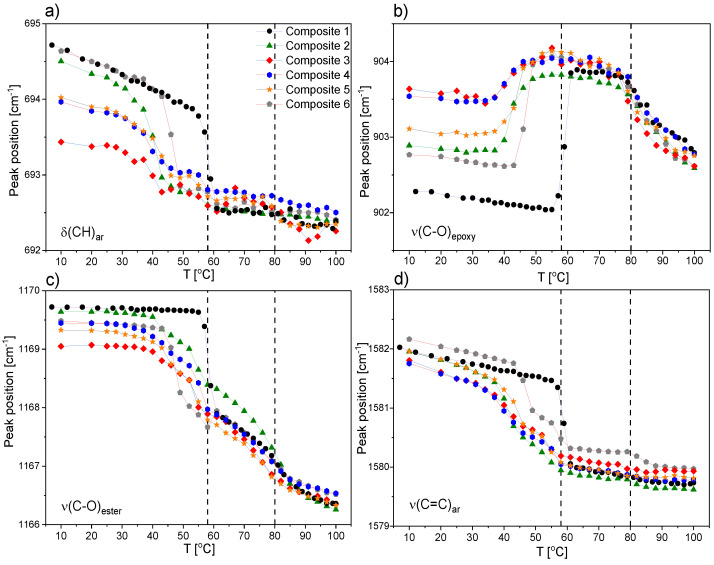
Temperature dependencies of peak maximum position for four selected IR bands connected to δ(CH)_ar_ (**a**), ν(C-O)_epoxy_ (**b**), ν(C-O)_ester_ (**c**), and ν(C=C)_ar_ (**d**) obtained for Composites 1–6 during cooling from the isotropic phase. The legend in graph (**a**) is the same for all graphs. The vertical dashed and dotted lines represent the SmA*–SmC* and SmC*–Cr_1_ phase transition temperatures (during cooling) for pure EHPDB, respectively.

Figure 19 presents temperature evolutions of the peak maximum position of four bands arising from the vibrational modes within aromatic rings and epoxy and ester groups. The vertical arrows denote phase transitions temperatures observed for pure EHPDB at about 80 and 58 °C during cooling. The first transition observed with this spectroscopic technique was connected to the N*–TGBA*, whereas the second one was connected to the SmC*–Cr transition. The higher temperature transition was gently accented by a small rise in the peak position values, and was visible at about the same temperature region for all six studied composites. The lower temperature transition to the ordered crystal phase was clearly visible when shifting the band towards larger wavenumbers in the case of all results, except for the band related to the epoxy group (Figure 19b), where we observed the opposite effect—shifting towards smaller wavenumbers. Spectroscopic results confirmed that this transition occurred at a significantly higher temperature for pure EHPDB in comparison to the composites with the *γ*-Fe_2_O_3_ admixture. Comparing the results shown in Figure 19a,b, it can be noted that at 100 °C, the peak positions of both bands for all six investigated composites were more or less the same. However, this changed after cooling samples to 10 °C, where differences in positions were obvious. Considering the band associated with the stretching mode of epoxy group, the lowest value of the peak position was observed for pure EHPDB. Then, for composites with nanoparticles, it shifted to a larger wavenumber in the following order: Composite 6, Composite 2, Composite 5, Composite 4, Composite 3. This could be explained by the fact that generally, the higher the concentration of nanoparticles, the more distorted the molecular structure, with weaker interactions of epoxy groups. Molecules in pure EHPDB were better aligned, and thus the interactions of epoxy groups were stronger, which was visible in the IR spectrum by the lower wavenumber (higher wavelength, lower energy) at which the IR stretching band appeared. Exactly the opposite order in the position of the band associated with the bending mode within the aromatic ring can be observed in Figure 19a.

Taking into consideration the results obtained for the band related to the ν(C=C)_ar_ mode, it was clear that for pure EHPDB, the transition to an ordered crystal phase was the sharpest, and occurred in one step (Figure 19d). In turn, the shape of the curves in the vicinity of the SmC*–Cr transition region for all the other composites with admixture of *γ*-Fe_2_O_3_ was different. As can be seen, the conversion to this phase occurred in two steps (two distinguishable increases in the value of peak position were visible), which may have indicated a slightly different mechanism of this transition; for example, through an intermediate state. The same two-step increase in the peak position value at P(T) for composites with *γ*-Fe_2_O_3_ also could be observed for the band connected to the ester group, but it was less pronounced (see Figure 19c).

**Figure 20:**
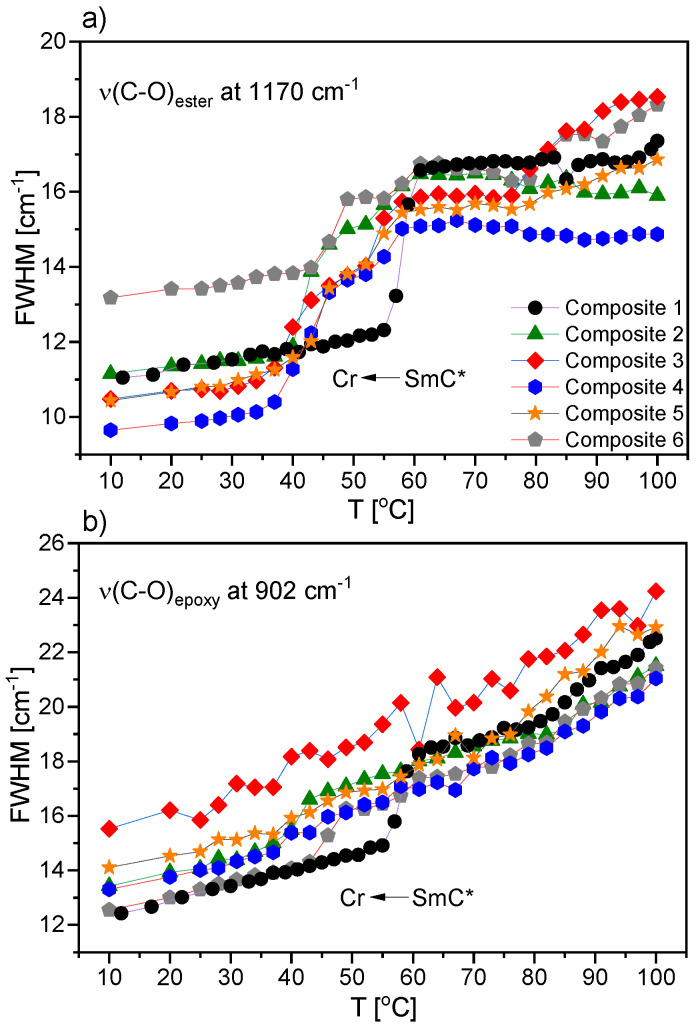
Temperature dependencies of the full width at half maximum (FWHM) of two selected IR bands connected to ν(C-O)_ester_ (**a**) and ν(C-O)_epoxy_ (**b**) modes obtained for pure EHPDB and nanocomposites during cooling from the isotropic phase. The positions of these two bands are given for pure EHPDB at room temperature. The legend in graph (**a**) is the same for all graphs.

Figure 20 presents the temperature dependencies of the full width at half maximum (FWHM) of two bands connected to the stretching modes of ester and epoxy group. This band parameter was very sensitive to molecular dynamics and ordering of the investigated group. Taking into consideration the results in Figure 20a obtained for the band associated with the ester group, it was clear that for pure EHPDB, the transition to ordered crystal phase was the sharpest, and occurred in one step, whereas for all the other composites with admixture of *γ*-Fe_2_O_3_, it occurred in two steps (two distinguishable declines in FWHM value were visible). These results were very consistent with the ones previously described (Figure 19c,d).

Figure 20b shows the temperature dependencies of FWHM for the band associated with the stretching mode of the epoxy group. These results showed that for pure EHPDB and Composites 2 and 6, the transition to the ordered crystal phase was sharper. In the cases of composites with a larger amount of nanoparticles (0.5 wt %, 0.7 wt %, and 0.9 wt % *γ*-Fe_2_O_3_), no distinct decline in FWHM value was observed at FWHM(T). The FWHM value decreased gradually in the entire investigated temperature range, which may suggest that the structure was less ordered in the low-temperature range for Composites 3–5 in comparison to Composites 1, 2, and 6. Moreover, at higher temperatures in liquid crystalline phases, the arrangement of molecules was more distorted, and hence the transition to the ordered crystal phase occurred in a less cooperative way, which also caused a decrease in the phase transition temperature.

**Figure 21:**
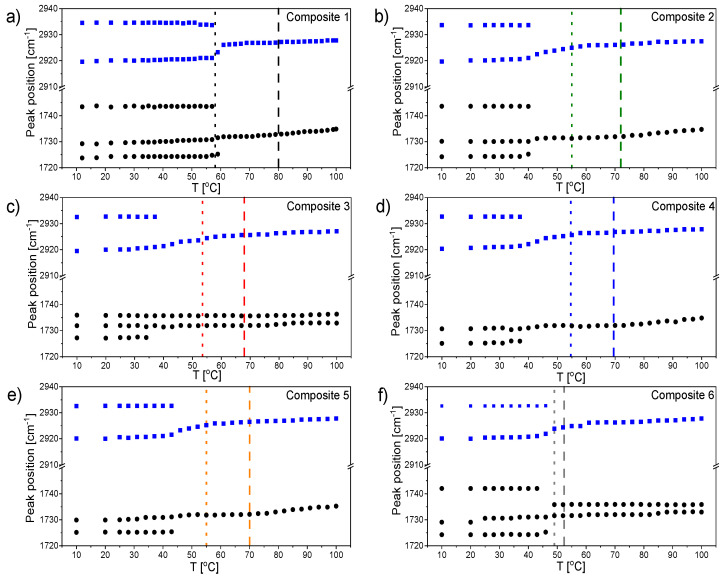
Temperature dependencies of the peak maximum position of two IR bands connected to ν(C=O) (black circles) and ν_as_(CH_2_) (blue squares) modes obtained for pure EHPDB—Composite 1 (**a**), Composite 2 (**b**), Composite 3 (**c**), Composite 4 (**d**), Composite 5 (**e**), and Composite 6 (**f**) during cooling from the isotropic phase. The vertical dashed and dotted lines represent the SmA*–SmC* and SmC*–Cr_1_ phase transition temperatures obtained during cooling by DSC, respectively.

Figure 21 presents the temperature dependencies of the peak maximum position of two IR bands associated with the ν(C=O) and ν_as_(CH_2_) stretching modes, upon doping with various amounts of nanoparticles, in the investigated system. Infrared vibrations of carbonyl group and CH_2_ methylene groups within hydrocarbon side chain were very sensitive to a change in molecular interactions. In the case of all six investigated composites, the transition from the SmC* to Cr phase was clearly visible. Both parts of the EHPDB molecule, the carbonyl group and hydrocarbon chain, were involved in this transition. 

The thermal behavior of the ν_as_(CH_2_) band was identical for all six systems. At 100 °C, it appeared at about 2928 cm^−1^, which proved that a highly disordered ’liquidlike’ material was formed. The position of this band almost did not change in a wide range of temperatures. This suggested that also in the N*, TGBA*, SmA*, and SmC* phases, the hydrocarbon chain was completely melted. At the SmC*–Cr phase transition, it split into two components, which was a result of interactions of molecules in a unit cell. In turn, the thermal behavior of the ν(C=O) band was more complex, and differed depending on the admixture concentration, which proved that *γ*-Fe_2_O_3_ nanoparticles modified the EHPDB matrix. For Composite 1 (pure EHPDB), there was one maximum observed in the liquid crystalline phases that corresponded well to one type of carbonyl group interacting in the same way with the environment. At the transition to the ordered crystal phase, the band split into three components, which indicated that three different subpopulations were present in the molecular structure. One shoulder was visible at a larger wavenumber at about 1743 cm^−1^, which could be attributed to the carbonyl group freely vibrating, and thus not being involved in the intermolecular interactions. This type of carbonyl group was also observed for Composites 2 and 6, and thus with the lowest amount of nanoparticles. The other two components, with maxima at about 1732 and 1725 cm^−1^, most probably were connected with two types of carbonyl groups involved in the intermolecular interactions, which were of a different nature. In the case of the compositions with a larger admixture of *γ*-Fe_2_O_3_ nanoparticles, Composites 4–5, temperature evolutions of positions of both investigated bands were identical—they both split into two components at P(T). Interestingly, at high temperatures for Composites 3 and 6, the ν(C=O) band was not fully symmetrical, and two components of this band could be distinguished.

## 3. Materials and Methods

### 3.1. Materials

The pure EHPDB compound and *γ*-Fe_2_O_3_ nanoparticles (diameter <50 nm) are commercially available, and were purchased in Sigma Aldrich (Sigma Aldrich Co., Saint Louis, MO, USA). According to the manufacturer, the purity of LC matrix—EHPDB was 98% (product number 328537), while the purity of the NPs was not given (product number 544884).

### 3.2. UV-Vis (Ultraviolet-Visible Spectroscopy)

Solid state UV-vis/NIR absorption spectra for Samples 1–2 and Composites 1–6 were recorded in reflectance mode using a JASCO MSV-370 microUV-vis/NIR spectrometer (JASCO Corporation, Tokyo, Japan) equipped with a LINKAM LTS420 hot stage with a programmable T96 system controller and an LNP96 cooling option under flow of dry nitrogen gas (Linkam Scientific Instruments Ltd., Epsom, England), for solid samples spread on the surface of a 0.5 mm thick quartz plate mounted with Kapton tape on a heating plate. The measurements were conducted in the temperature range of 0–120 °C at a rate of 2 °C/min. Each 4 min spectrum measurement was preceded by a 2 min waiting time for temperature stabilization.

### 3.3. FDDS (Frequency Domain Dielectric Spectroscopy)

In order to determine the dielectric properties of the new synthesized nanocomposites, a Turnkey Impedance Spectrometer Concept 81 (Novocontrol Technologies GmbH & Co. KG, Montabaur, Germany) was used. Measurements were carried out by using planar cells with gold-coated electrodes (ca. C_0_ = 50 pF, active area 25 mm^2^, AWAT Company, Warsaw, Poland) in the frequency range of 0.5–10^6^ Hz (measuring voltage 0.5 V). ITO cells were not used to eliminate a parasitic relaxation process around 1MHz (coming from indium tin oxide, a very pronounced process often covers/overlaps the relaxation process of studied materials), while parasitic relaxation coming from the Au electrodes was outside of the measuring frequency range [91]. The composites were introduced into the measuring cells due to the capillary effect at a temperature slightly above the clearing point, and slowly cooled down. Due to the planar alignment of the liquid crystal molecules in the cells, the perpendicular component of the dielectric permittivity was measured [92]. The measurements were taken during cooling from 110.0 to 25.0 °C with a 1.0 °C temperature interval.

### 3.4. SQUID (Superconducting Quantum Interference Device Magnetometry)

Magnetic measurements were performed on Samples 1–2 of about 1 mg and Composites 1–6 of about 10 mg. The samples were placed at the bottom of gelatin capsules (approximately 30 mg), blocked with nitrocellulose varnish (approximately 20 mg after drying), and quartz wool (10–15 mg). Next, capsules were placed inside Kapton tubes and mounted on a magnetic test rod. Measurements were conducted by using a Quantum Design MPMS XL magnetometer (Quantum Design Inc., San Diego, CA, USA). All direct-current (DC) measurements were corrected for the diamagnetic contribution of the sample holder and the EHPDB liquid crystal matrix.

### 3.5. FT-MIR (Fourier Transform Mid-Infrared Spectroscopy)

Temperature-dependent Fourier transform mid-infrared (FT-MIR) absorption measurements were performed in transmission mode using a VERTEX 70v vacuum spectrometer (Bruker, Billerica, MA, USA) for bulk samples of Composites 1–6. More details on this experiment can be found in our recent paper [39]. The band parameters (peak maximum position and FWHM) of chosen bands at different temperatures were obtained by fitting the Lorentz function using OriginPro, Version 2020 (OriginLab Corporation, Northampton, MA, USA).

## 4. Conclusions

We showed the influence of the *γ*-Fe_2_O_3_ nanoparticle admixture, as well as of oleic acid, on the physical properties of the ferroelectric liquid crystal EHPDB by using light absorption in the UV-vis and MIR ranges, dielectric spectroscopy, and magnetic superconducting quantum interference device magnetometry methods.

It was found that both pure (Sample 1) and oleic-acid-decorated (Sample 2) *γ*-Fe_2_O_3_ nanoparticles absorbed in the 200–700 nm range with distinct absorption bands around 225, 350, 500, and 650 nm; however, the intensities of the absorption bands were smaller for Sample 2; while above 750 nm, both materials were transparent. All Composites 1–6 were completely transparent in the range of 500–850 nm, regardless of temperature, and the temperature dependence of the absorbance for selected wavelengths showed distinct discontinuities at the phase transition temperatures, with a visible plateau in the liquid crystalline ferroelectric SmC* and isotropic phases. In the absorption spectra of the nanocomposites (Composites 2–5), a decrease in the absorption in the UV range and the appearance of additional absorption bands in the 350–600 nm region compared to the pure LC matrix (Composite 1) were observed. In our opinion, nanoparticles in the organic matrix played the role of scattering centers of incident light, and with the increase in nanoparticle concentration, the intensity of the scattered light increased, and thus the absorption decreased.

The dielectric spectroscopy method allowed us to register and interpret relaxation processes appearing in the liquid crystalline and crystal phases in Composites 1–5. Two relaxation processes in the liquid crystalline N* phase were revealed, namely MWS and the molecular s-process. The observed decrease in the relaxation frequency of the s-process in nanocomposites was related to the presence of nanoparticles in the LC matrix, which hindered molecular reorientation (molecules in Composites 2–5 needed a longer time for reorientation). In turn, in the liquid crystalline TGBA* and SmA* phases, in addition to the MWS process, a soft mode was registered (with the exception of Composite 2) in a narrow temperature range. In the liquid crystalline ferroelectric SmC* phase, the MWS process was still observed, and additionally, the Goldstone mode (however, for Composite 2, the GM was covered by the very strong MWS process). On the other hand, the origin of the additional relaxation process registered in this phase for Composites 4 and 5 was not fully explained. It may have been related to the movement of nanoparticles in the form of aggregates or the collective movement of nanoparticles (or aggregates) with the surrounding organic molecules, and the impurities (not ions) existing in the LC matrix. In addition, a strong modification of the Goldstone mode in the SmC* phase was observed (e.g., extension of the relaxation time by about 12 times) after introducing even the smallest amount of *γ*-Fe_2_O_3_ nanoparticle admixture, which, as already mentioned, may have been caused by difficulties in the reorientation of organic molecules in the presence of nanoparticles. In general, in the liquid crystalline phases, the doping caused an increase in the dielectric constant. In turn, in the crystal phase, the MWS process disappeared and a new one, most likely of a molecular nature, and weakly dependent on the concentration of *γ*-Fe_2_O_3_ nanoparticles, appeared. The specific conductivity increased over the entire frequency range with an increase in the *γ*-Fe_2_O_3_ nanoparticles’ concentration.

Based on the results obtained by the SQUID method, it was found that the blocking temperature of the used *γ*-Fe_2_O_3_ nanoparticles was above 380 K, and the nanoparticles retained their properties in the LC matrix; therefore, for Composites 2–5, the liquid crystalline ferroelectric SmC* phase coexisted with the magnetically blocked state of the nanoparticles. Evidence of the presence of nanoparticles in the blocked state in Composites 2–5 was the similar shape of the hysteresis loop, which narrowed with increasing temperature. Saturation magnetization decreased with increasing temperature, which was related to the higher thermal energy of nanoparticles. However, it turned out that the coercive field depended not only on temperature, but also on the concentration of nanoparticles. The coercive field was greater for Composites 2–5 than for pure nanoparticles (Sample 1); therefore, nanoparticles immersed in the LC matrix required a higher magnetic field to invert the magnetization (especially for Composite 3). The addition of oleic acid additionally increased the coercive field and saturation magnetization.

Infrared absorption spectroscopy results showed that addition of the *γ*-Fe_2_O_3_ nanoparticles had an influence on the SmC*–Cr phase transition temperature of liquid crystal EHPDB, and moderated its molecular arrangement. Parts of the EHPDB molecule containing oxygen, such as carbonyl, ester, and epoxy groups, as well as aromatic rings, were sensitive to changes in intermolecular interactions and/or dynamics due to the presence of nanoparticles.

To sum up, oleic acid used to decorate nanoparticles, on the one hand, prevented the agglomeration of nanoparticles, causing better homogeneity in the LC matrix, which in turn increased the coercive field and the M_R_, while decreasing the M_s_ and strongly modifying the temperature of phase transitions (narrowing the mesophases). It also influenced the absorption of light in the UV-vis range in the nanocomposites. However, further studies of the effect of oleic acid (and other surfactants) on the characteristic parameters of organometallic nanocomposites, with particular emphasis on the amount of surfactant used (at a constant concentration of nanoparticles), seem necessary.

## Figures and Tables

**Table 1:** Predicted and estimated weight percentages of *γ*-Fe_2_O_3_ nanoparticles in the samples.

Sample/Composite	Predicted wt % of *γ*-Fe_2_O_3_	Estimated wt % of *γ*-Fe_2_O_3_
Sample 1	100	100
Sample 2	100	11.9(1)
Composite 2	0.3	0.13(6)
Composite 3	0.5	0.37(5)
Composite 4	0.7	0.60(5)
Composite 5	0.9	0.75(4)
Composite 6	0.3	0.16(5)

**Table 2:** Magnetic properties of Samples 1–2 and Composites 2–6. Average M_R_ and M_S_ were normalized by taking the concentration of *γ*-Fe_2_O_3_.

Sample/ Composite	T[K]	Aver. H_c_ (Oe)	Aver. M_S_ (emu/g)	Aver. M_R_ (emu/g)	Aver. M_R_/M_S_
Sample 1	300	133.5	66.20	13.56	0.205
	200	179.6	70.76	17.06	0.241
	100	240.9	72.83	21.18	0.291
	10	335.0	73.29	27.16	0.371
Sample 2	300	152.3	65.60	14.98	0.228
	200	200.9	70.48	18.90	0.268
	100	260.4	72.16	22.82	0.316
	10	352.1	71.97	28.36	0.394
Composite 2	300	128.7	66.65	14.74	0.221
	200	185.6	69.69	21.68	0.311
	100	242.0	71.75	25.01	0.349
	10	294.5	72.46	25.98	0.359
Composite 3	300	154.3	67.29	16.86	0.251
	200	213.9	71.78	22.14	0.308
	100	269.7	72.66	27.79	0.382
	10	330.3	73.16	28.98	0.396
Composite 4	300	147.2	66.87	15.20	0.227
	200	209.3	71.56	22.48	0.314
	100	267.8	73.63	27.45	0.373
	10	330.6	73.45	28.26	0.385
Composite 5	300	145.9	65.98	15.01	0.227
	200	201.3	70.53	22.64	0.321
	100	258.9	72.49	26.71	0.368
	10	330.4	73.90	27.38	0.371
Composite 6	300	165.6	67.38	16.75	0.249
	200	225.7	70.52	24.06	0.341
	100	277.8	72.41	28.39	0.392
	10	344.1	72.44	29.28	0.404

## Data Availability

Not applicable.

## Supplementary Materials

The following are available online at https://www.mdpi.com/article/10.3390/ijms23010050/s1.
