# Supplementary material for: Modifications of EHPDB Physical Properties through Doping with Fe2O3 Nanoparticles (Part II)
Source: Int J Mol Sci. 2021 Dec 21;23(1):50. doi: 10.3390/ijms23010050 (PMC8744552; doi:10.3390/ijms23010050)
Supplement: Supplementary file 1 [file ijms-23-00050-s001.zip › ijms-1491258-supplementary.pdf]

Supplementary Material

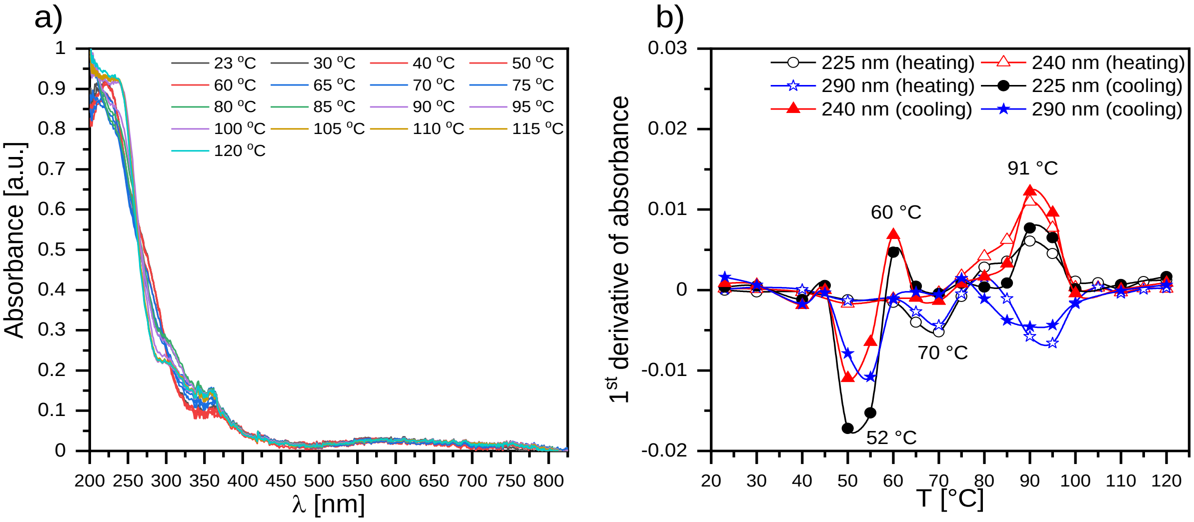

Figure S1. Solid state UV-Vis absorption spectra of Composite 1 (pure EHPDB) at several chosen temperatures in the 200–850 nm range during heating (**a**) and temperature dependence of the first derivative of the absorbance for selected wavelengths 225, 240 and 290 nm during heating and cooling (**b**).

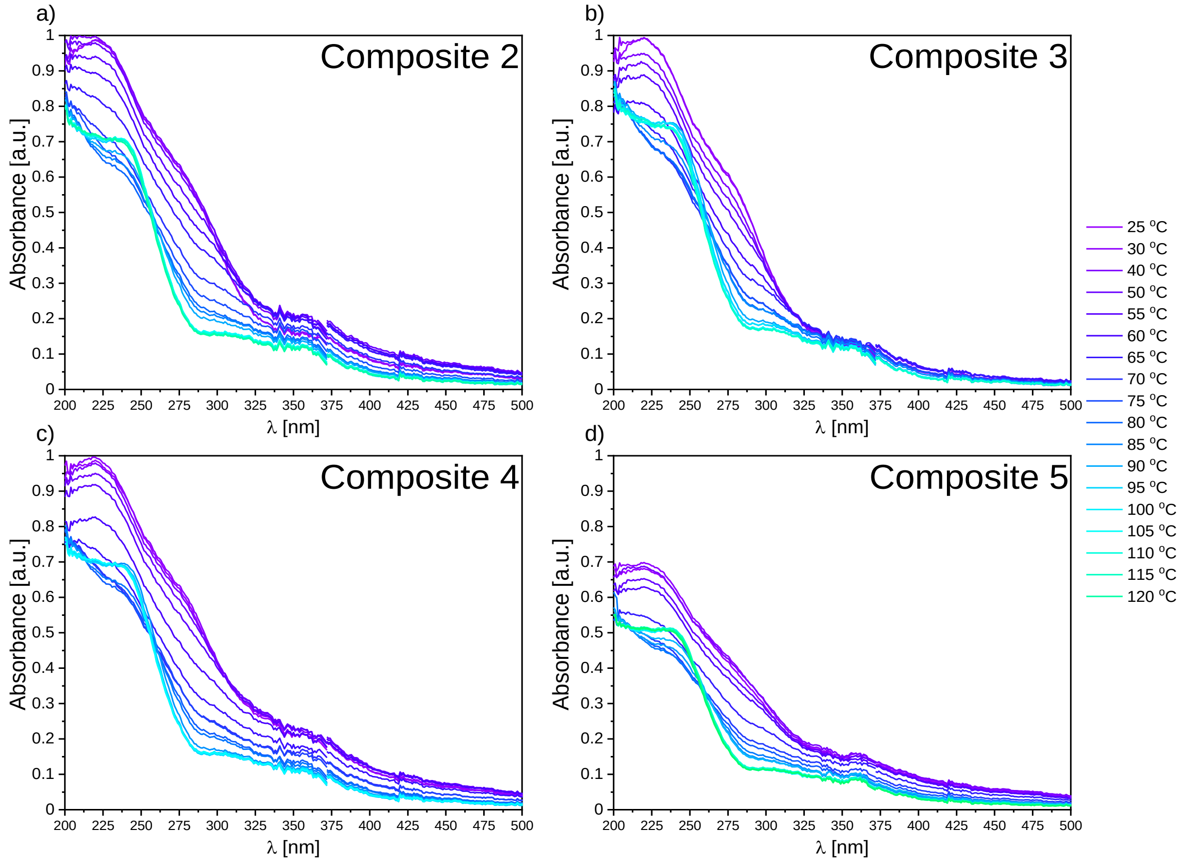

Figure S2. Solid state UV-Vis absorption spectra of Composites 2–5 at several chosen temperatures in the 200–500 nm range during heating. The legend from the right side is for all graphs.

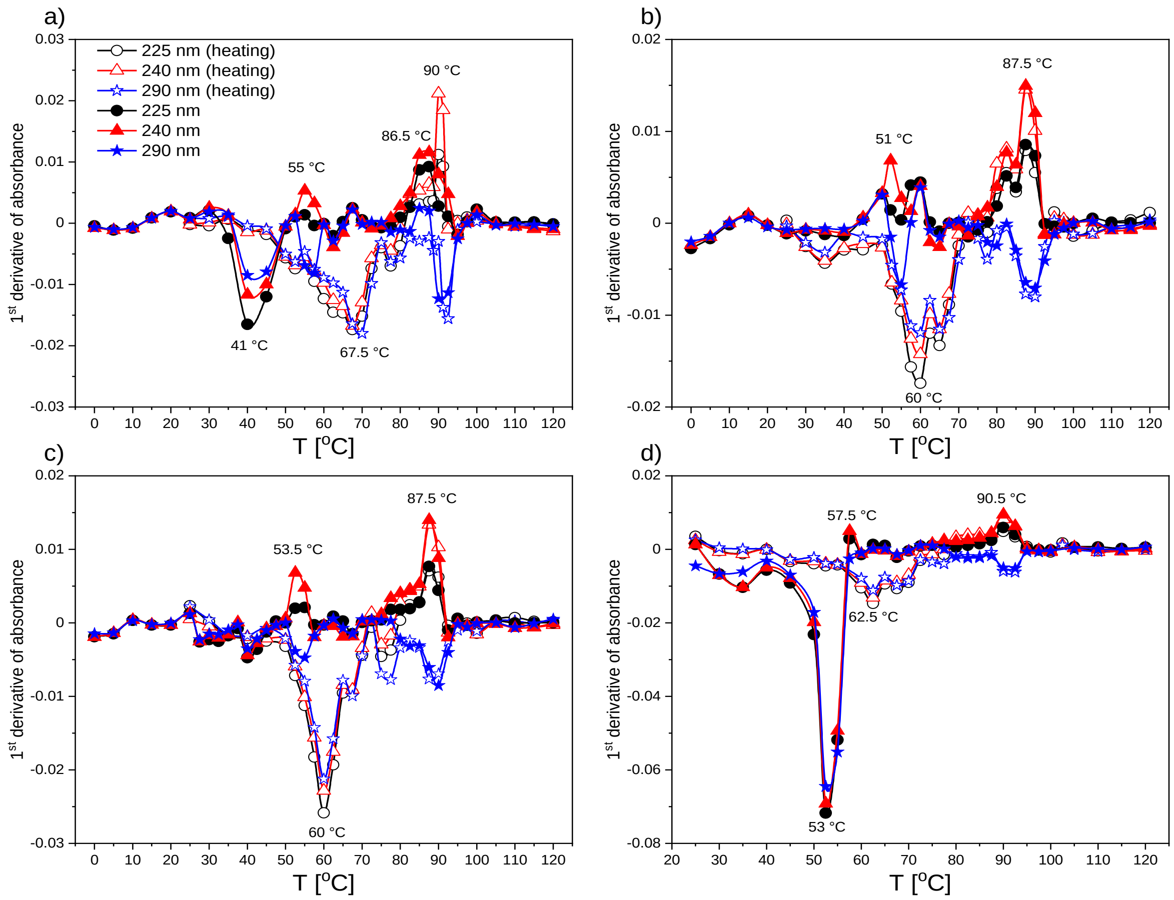

Figure S3. Temperature dependence of the first absorbance derivative for selected wavelengths (225, 240, 290 nm) for Composites 2–4 during heating and cooling. The given temperatures correspond to the phase transitions temperatures.

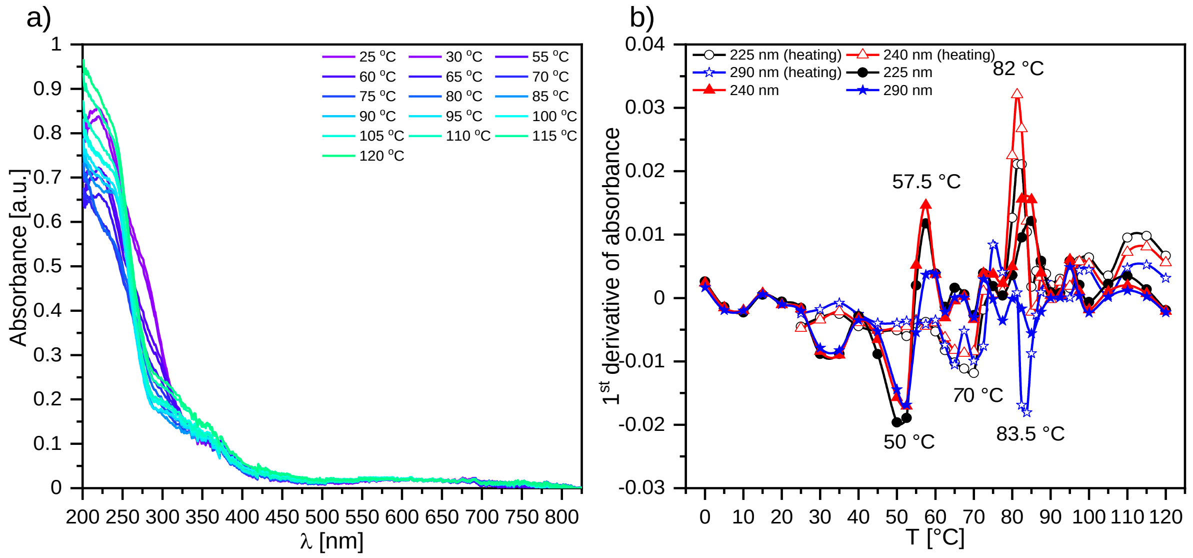

Figure S4. Solid state UV-Vis absorption spectra of Composite 6 at several chosen temperatures in the 200–850 nm range during heating (**a**) and temperature dependence of the first derivative of absorbance for selected wavelengths 225, 240 and 290 nm during heating and cooling (**b**).

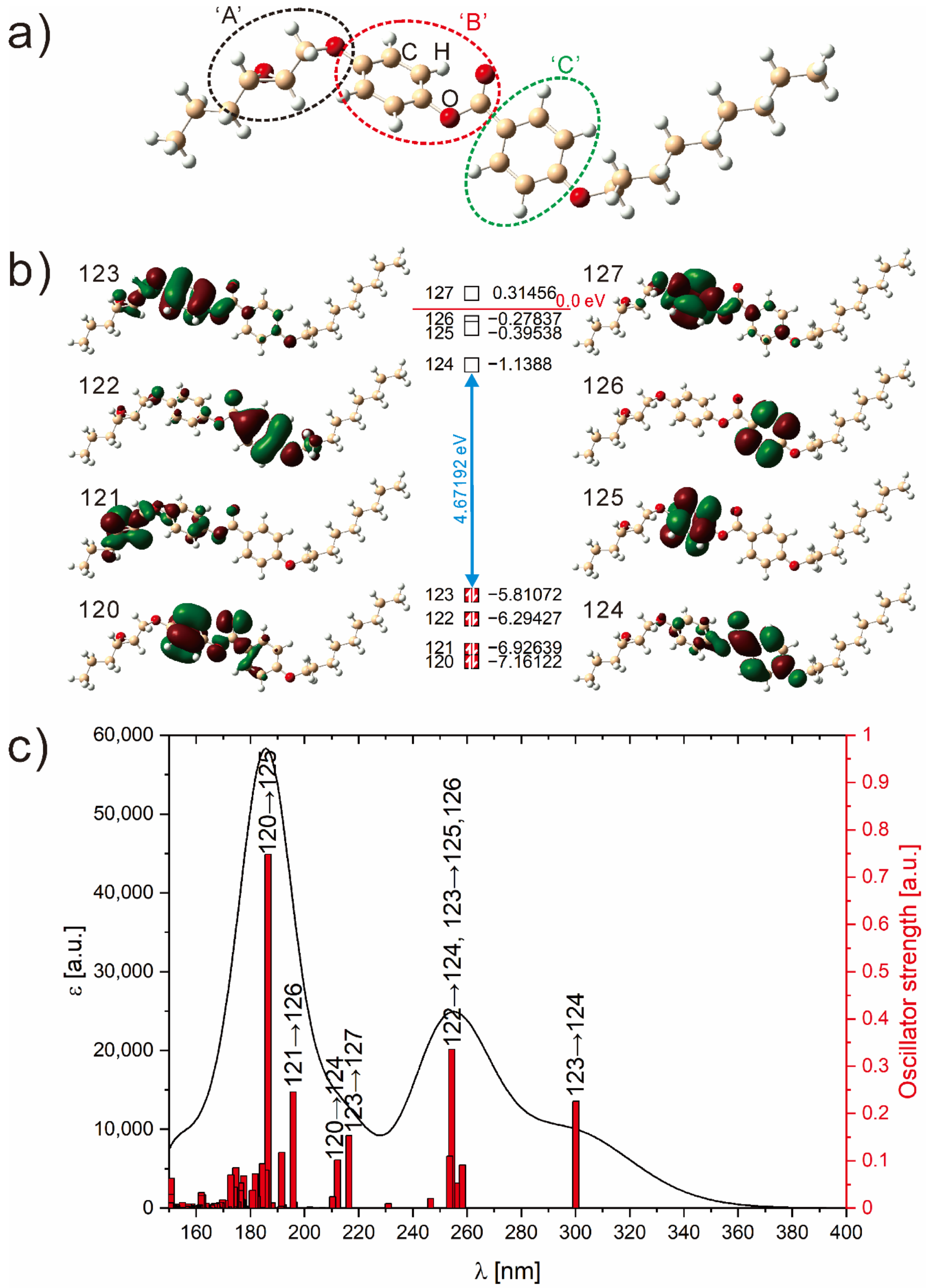

Figure S5. Model for optimized structure of EHPDB molecule used for calculation (**a**). Energy level diagram in eVs with calculated frontier molecular orbitals (**b**). Blue arrow and number correspond to the HOMO-LUMO gap. Calculated UV-Vis spectrum for optimized structure of EHPDB molecule (**c**). Red bars indicate electronic transitions with the largest contributions while legend above bars indicate electronic states involved in transition.

Table S1. List of atomic coordinates for optimized structure of EHPDB molecule.

Atom	X	Y	Z	Atom	X	Y	Z
C	2.1355	−1.7731	1.7295	H	18.7163	−6.4138	3.3196
C	16.9662	−6.67	1.4823	H	19.5618	−7.2845	0.4477
O	17.5989	−7.9895	1.7894	H	19.3947	−5.4226	0.8071
C	18.3242	−6.7769	2.2839	H	15.8922	−7.0283	3.4813
C	19.539	−6.4753	1.2862	H	15.6587	−5.2811	2.7627
C	15.7528	−6.413	2.5014	H	20.8657	−5.7703	2.9961
C	20.9204	−6.5073	2.0949	H	21.1219	−7.5865	2.4865
C	22.0976	−6.0631	1.1035	H	21.7464	−6.1956	0
O	14.479	−6.8472	1.8596	H	22.3645	−4.9446	1.2944
C	13.2898	−6.0524	1.8825	H	23.0369	−6.7264	1.2944
C	11.9971	−6.7122	1.9612	H	11.9392	−7.8749	2.019
C	13.3565	-4.5992	1.8143	H	14.3911	−4.0658	1.7538
C	10.7765	−5.9258	1.9678	H	9.7435	−6.4618	2.0306
C	12.1328	−3.8114	1.8217	H	12.1885	−2.6484	1.7677
C	10.8396	−4.4732	1.8963	H	5.7912	−5.2256	2.37
O	9.6371	−3.6925	1.8897	H	8.1924	−1.381	2.2373
C	8.4122	−4.1925	2.4323	H	3.578	−3.8497	2.1924
O	8.393	−5.3253	3.007	H	5.9759	0	2.0545
C	7.1139	−3.3776	2.3165	H	2.3423	−2.4405	3.8919
C	5.8328	−4.0628	2.3017	H	0.7655	−1.4437	3.5125
C	7.1637	-1.9288	2.2274	H	0	−3.822	3.8548
C	4.6036	−3.2961	2.2017	H	4E-4	−3.5521	1.9704
C	5.9322	−1.1625	2.1258	H	2.2389	−4.7767	1.7185
C	4.6462	−1.8431	2.1131	H	2.4766	−4.8496	3.6058
O	3.4319	-1.0871	2.0103	H	0.1743	−6.2538	3.722
C	1.4898	−2.2664	3.1164	H	0.2418	−6.361	1.8226
C	0.6709	−3.6365	2.92	H	2.5102	−7.5705	1.9061
C	1.6923	−4.868	2.7438	H	2.6407	−7.2993	3.7857
C	0.878	−6.251	2.7929	H	0.4021	−8.6975	4.0916
C	1.9063	−7.4789	2.8987	H	0.4111	−9.0525	2.2214
C	1.0777	−8.8291	3.151	H	2.7229	−10.226	2.4276
C	2.0861	−10.0534	3.3884	H	2.7911	−9.81	4.284
C	1.2389	−11.3758	3.7158	H	0.913	−11.8846	2.719
H	1.435	−1.0144	1.189	H	1.8962	−12.115	4.3324
H	2.3498	−2.6756	1.0239	H	0.2926	−11.0877	4.3324
H	16.6109	−6.1354	0.5095				

Table S2. List of calculated the first 25 excited states with oscillator strengths (f) for optimized structure of EHPDB molecule.

The Excited State	Transition	ΔE [eV], λ [nm], Oscillator Strength [a.u.]
1	123 -> 124 0.70335	Singlet-A, 4.1305 eV 300.17 nm f = 0.2260 <S**2≥0.000
2	118 -> 124 −0.27413<br>121 -> 124 −0.36861<br>121 -> 127 0.15271<br>122 -> 124 0.23654<br>123 -> 126 0.44480	Singlet-A, 4.8001 eV 258.30 nm f = 0.0911 <S**2≥0.000
3	120 -> 124 −0.34653<br>122 -> 124 −0.25683<br>122 -> 125 0.32247<br>123 -> 125 0.43452	Singlet-A, 4.8415 eV 256.09 nm f = 0.0528 <S**2≥0.000
4	118 -> 124 −0.15895<br>120 -> 124 −0.15580<br>121 -> 127 −0.12444<br>122 -> 124 0.50773<br>122 -> 125 0.16938<br>123 -> 125 0.10294<br>123 -> 126 −0.34003	Singlet-A, 4.8737 eV 254.39 nm f = 0.3359 <S**2≥0.000
5	118 -> 124 0.45562<br>121 -> 124 0.24850<br>121 -> 127 0.10203<br>122 -> 124 0.29195<br>123 -> 126 0.30331	Singlet-A, 4.8894 eV 253.58 nm f = 0.1100 <S**2≥0.000
6	120 -> 124 0.25940<br>122 -> 125 −0.37178<br>123 -> 125 0.53377	Singlet-A, 5.0289 eV 246.54 nm f = 0.0202 <S**2≥ 0.000
7	118 -> 124 −0.40403<br>121 -> 124 0.53980<br>123 -> 126 0.16477	Singlet-A, 5.3687 eV 230.94 nm f = 0.0098 <S**2≥0.000
8	122 -> 126 0.70133	Singlet-A 5.5626 eV 222.89 nm f = 0.0002 <S**2≥0.000
9	121 -> 126 −0.28317<br>123 -> 127 0.62473	Singlet-A, 5.7308 eV 216.35 nm f = 0.1536 <S**2≥0.000
10	119 -> 124 0.24260<br>120 -> 124 0.48546<br>122 -> 125 0.40384	Singlet-A, 5.8430 eV 212.19 nm f = 0.1021 <S**2≥0.000
11	119 -> 124 0.65286<br>120 -> 124 −0.15430<br>122 -> 125 −0.17068	Singlet-A, 5.8942 eV 210.35 nm f = 0.0240 <S**2≥0.000
12	118 ->125 0.27758<br>121 ->125 0.64849	Singlet-A, 6.0051 eV 206.46 nm f = 0.0013 <S**2≥0.000
13	120 -> 125 0.11877<br>122 -> 127 0.68693	Singlet-A 6.1379 eV 202.00 nm f = 0.0024 <S**2≥0.000
14	118 ->125 0.62777<br>121 ->125 −0.27155	Singlet-A, 6.3183 eV 196.23 nm f = 0.0122 < S**2≥0.000
15	118 -> 125 −0.12436<br>118 -> 126 0.55308	Singlet-A, 6.3308 eV 195.84 nm f = 0.2456 <S**2≥0.000

	121 -> 126	0.31534	
	123 -> 127	0.13476	
16	119 -> 126	0.37406	Singlet-A, 6.4302 eV 192.81 nm f = 0.0009 <S**2≥0.000
	120 -> 126	0.56814	
	121 -> 127	−0.11596	
17	119 -> 126	0.41361	Singlet-A, 6.4543 eV 192.09 nm f = 0.0068 <S**2≥0.000
	120 -> 126	−0.38587	
	121 -> 127	−0.16811	
	123 -> 129	0.31762	
	123 -> 131	0.12462	
18	116 -> 124	0.23025	Singlet-A, 6.4716 eV 191.58 nm f = 0.1179 <S**2≥0.000
	117 -> 124	0.59557	
	121 -> 126	0.14132	
	123 -> 129	−0.15735	
19	117 -> 124	0.13988	Singlet-A, 6.4811 eV 191.30 nm f = 0.0034 <S**2≥0.000
	118 -> 126	−0.13327	
	119 -> 126	−0.34348	
	120 -> 126	0.12315	
	123 -> 129	0.49678	
	123 -> 131	0.19739	
	123 -> 133	0.10558	
20	122 -> 128	0.37823	Singlet-A, 6.5932 eV 188.05 nm f = 0.0112 <S** 2≥0.000
	123 -> 128	0.55861	
21	116 -> 124	0.20560	Singlet-A, 6.6481 eV 186.50 nm f = 0.7486 <S**2≥0.000
	117 -> 124	−0.21538	
	118 -> 126	−0.23947	
	119 -> 125	0.21078	
	120 -> 125	0.43376	
	121 -> 126	0.22520	
22	116 -> 124	−0.11393	Singlet-A, 6.6743 eV 185.76 nm f = 0.0803 <S**2≥0.000
	119 -> 125	0.65434	
	121 -> 126	−0.10887	
23	112 -> 124	0.19937	Singlet-A, 6.7207 eV 184.48 nm f = 0.0936 <S**2≥0.000
	116 -> 124	0.46500	
	117 -> 124	−0.17961	
	118 -> 127	−0.15722	
	120 -> 125	−0.23204	
	121 -> 127	−0.16804	
	122 -> 128	0.13126	
	123 -> 133	−0.13578	
24	107 -> 124	0.15305	Singlet-A, 6.7372 eV 184.03 nm f = 0.0015 <S**2≥0.000
	109 -> 124	0.26722	
	110 -> 124	−0.11988	
	111 -> 124	0.19258	
	112 -> 124	0.37539	
	115 -> 124	−0.33331	
	116 -> 124	−0.15318	
	117 -> 124	0.10588	
	120 -> 125	0.10294	
25	118 -> 127	0.19228	Singlet-A, 6.7947 eV 182.47 nm f = 0.0245 <S**2≥0.000
	121 -> 127	0.21709	
	122 -> 128	0.48744	

	123 -> 128 −0.34414	

Table S3. List of atomic coordinates for 'planar' EHPDB molecule.

Atom	X	Y	Z	Atom	X	Y	Z
C	5.47221	−2.5107	−0.60355	H	−10.23004	1.17922	1.29781
C	−8.36095	0.6968	0.22883	H	−9.8744	−1.50468	−0.21298
O	−9.40905	1.10694	−0.66462	H	−10.40326	−1.35721	1.47605
C	−9.77589	0.48124	0.57779	H	−8.21632	2.67209	1.02497
C	−10.44645	−0.8687	0.48434	H	−7.16712	1.48542	1.85367
C	−7.5601	1.80383	0.87121	H	−12.47003	−0.12974	0.71867
C	−11.9042	−0.76885	0.01581	H	−11.92316	−0.24832	−0.9574
C	−12.58935	−2.13099	−0.10555	H	−12.06563	−2.77879	−0.82896
O	−6.50586	2.28139	0.03726	H	−12.60462	−2.66137	0.86213
C	−5.30563	1.62408	0.01339	H	−13.63213	−2.02738	-0.44545
C	−4.32343	2.20238	−0.80822	H	−4.58109	3.10741	−1.36065
C	−4.99838	0.45943	0.73022	H	−5.73375	−0.02747	1.36993
C	−3.05488	1.64129	−0.91316	H	−2.30108	2.10473	−1.54657
C	−3.71964	−0.10115	0.62848	H	−3.46885	−1.00818	1.181
C	−2.75459	0.48314	−0.18589	H	2.19216	1.1939	−0.12605
O	−1.53805	−0.19479	−0.29163	H	−0.32046	−2.2673	−0.55797
C	−0.34738	0.48732	−0.21758	H	4.21594	−0.22473	−0.28934
O	−0.26804	1.68749	−0.07975	H	1.71232	−3.71281	−0.73686
C	0.80844	−0.43497	−0.33055	H	5.42346	−1.59827	1.36278
C	2.09576	0.11564	−0.26092	H	5.76511	−3.33185	1.34584
C	0.67427	−1.82605	−0.50097	H	7.8058	−2.19416	1.97041
C	3.2316	−0.68624	−0.35595	H	8.05161	−2.86737	0.36064
C	1.79831	−2.63362	−0.6002	H	7.65893	−0.59086	−0.65686
C	3.08899	−2.0743	−0.53074	H	7.32997	0.0699	0.94753
O	4.111	−2.96026	−0.65428	H	9.67135	−0.5212	1.66507
C	5.98795	−2.383	0.8297	H	9.99448	−1.19774	0.06735
C	7.4936	−2.09	0.91643	H	9.5793	1.06237	−0.97497
C	7.91473	−0.7027	0.41286	H	9.25435	1.73951	0.62222
C	9.41002	−0.42028	0.59476	H	11.59711	1.14814	1.34974
C	9.84067	0.96285	0.09565	H	11.92195	0.46959	-0.24711
C	11.33503	1.24648	0.27903	H	11.50415	2.72691	−1.29083
C	11.76613	2.6294	−0.22093	H	11.17939	3.40508	0.30521
C	13.26003	2.90345	−0.03344	H	13.87388	2.16538	−0.57767
H	6.03464	−3.29306	−1.13621	H	13.5376	3.90409	−0.40215
H	5.58344	−1.57559	−1.17684	H	13.54658	2.84885	1.0308
H	−7.78272	−0.16538	−0.12775				

Table S4. List of calculated the first 25 excited states with oscillator strengths (f) for 'planar' EHPDB molecule.

The Excited State	Transition	ΔE [eV], λ [nm],<br>Oscillator Strength [a.u.]
1	123 -> 124 0.70328	Singlet-A, 3.7065 eV 334.50 nm f = 0.2535 <S**2≥0.000
2	117 -> 124 −0.15514<br>118 -> 124 0.15592<br>119 -> 124 −0.35303<br>120 -> 124 0.12629<br>121 -> 124 −0.35605<br>122 -> 124 0.39191	Singlet-A, 4.1199 eV 300.94 nm f = 0.0739 <S**2≥0.000
3	120 -> 124 −0.31977<br>122 -> 125 0.28814	Singlet-A, 4.4230 eV 280.31 nm f = 0.0097 <S**2≥0.000

	123 -> 125	0.54506	
4	121 -> 124	−0.39317	Singlet-A, 4.4371 eV 279.42 nm f = 0.1201 <S**2≥0.000
	121 -> 127	0.16869	
	122 -> 124	−0.28707	
	123 -> 126	0.46020	
5	120 -> 124	0.33583	Singlet-A, 4.5315 eV 273.60 nm f = 0.0463 <S**2≥0.000
	122 -> 124	−0.24274	
	122 -> 125	−0.35422	
	123 -> 125	0.39292	
	123 -> 126	−0.14252	
6	118 -> 124	−0.11928	Singlet-A, 4.5857 eV 270.37 nm f = 0.2036 <S**2≥0.000
	119 -> 124	0.30990	
	120 -> 124	0.11627	
	121 -> 124	0.12768	
	121 -> 127	0.10476	
	122 -> 124	0.40008	
	122 -> 125	−0.20588	
	123 -> 125	0.20816	
	123 -> 126	0.26267	
7	117 -> 124	−0.12542	Singlet-A, 4.7730 eV 259.76 nm f = 0.0305 <S**2≥0.000
	118 -> 124	0.12843	
	119 -> 124	−0.32172	
	121 -> 124	0.43825	
	121 -> 127	0.16494	
	123 -> 126	0.33756	
8	121 -> 126	−0.23526	Singlet-A, 5.2327 eV 236.94 nm f = 0.1533 <S**2≥0.000
	122 -> 126	0.11869	
	123 -> 127	0.62160	
9	119 -> 125	0.13200	Singlet-A, 5.2791 eV 234.86 nm f = 0.0591 <S**2≥0.000
	120 -> 124	−0.35113	
	121 -> 125	0.40235	
	122 -> 125	−0.37373	
	123 -> 127	0.11784	
10	120 -> 124	0.30226	Singlet-A, 5.3602 eV 231.30 nm f = 0.0563 <S**2≥0.000
	121 -> 125	0.54606	
	122 -> 125	0.25095	
11	119 -> 126	−0.11192	Singlet-A, 5.3940 eV 229.86 nm f = 0.0277 <S**2≥0.000
	122 -> 126	0.66766	
	123 -> 127	−0.12259	
12	117 -> 124	−0.19378	Singlet-A, 5.4341 eV 228.16 nm f = 0.0031 <S**2≥0.000
	118 -> 124	0.58031	
	119 -> 124	0.32559	
13	115 -> 124	0.12820	Singlet-A, 5.5323 eV 224.11 nm f = 0.0036 <S**2≥0.000
	117 -> 124	0.60097	
	118 -> 124	0.30094	
	119 -> 124	−0.12426	
14	117 -> 125	0.15184	Singlet-A, 5.6847 eV 218.10 nm f = 0.0237 <S**2≥0.000
	118 -> 125	−0.19546	
	119 -> 125	0.50366	
	120 -> 125	−0.27682	
	121 -> 125	−0.16855	
	122 -> 127	−0.25127	

15	118 -> 125	−0.10816	Singlet-A, 5.7222 eV 216.67 nm f = 0.0032 <S**2≥0.000
	119 -> 125	0.29022	
	120 -> 125	0.11133	
	122 -> 127	0.59092	
16	116 -> 124	0.13723	Singlet-A, 5.8422 eV 212.22 nm f = 0.2235 <S**2≥0.000
	118 -> 126	−0.10566	
	119 -> 126	0.42070	
	120 -> 126	−0.17106	
	121 -> 126	0.39124	
	122 -> 127	−0.11257	
	123 -> 127	0.10427	
	123 ->128	−0.17375	
17	114 -> 124	0.10177	Singlet-A, 5.8828 eV 210.76 nm f = 0.0065 <S**2≥0.000
	116 -> 124	0.66601	
18	119 -> 126	0.25900	Singlet-A, 5.9471 eV 208.48 nm f = 0.0369 <S**2≥0.000
	120 -> 125	−0.11406	
	120 -> 126	−0.11429	
	121 -> 126	−0.10412	
	123 -> 128	0.57129	
	123 -> 130	0.11413	
19	119 -> 126	−0.15914	Singlet-A, 6.0095 eV 206.31 nm f = 0.0038 <S **2≥0.000
	120 -> 126	0.10989	
	121 -> 127	0.11700	
	123 -> 130	0.59928	
	123 -> 131	−0.16079	
	123 -> 138	−0.12987	
20	115 -> 124	0.34686	Singlet-A, 6.0471 eV 205.03 nm f = 0.0229 <S**2≥0.000
	117 -> 124	−0.11531	
	117 -> 126	−0.22305	
	118 -> 126	0.10840	
	119 -> 127	0.19956	
	120 -> 125	0.10035	
	121 -> 127	0.38777	
	123 -> 126	−0.13044	
	123 -> 130	−0.15059	
21	115 -> 124	0.39247	Singlet-A, 6.1044 eV 203.11 nm f = 0.4016 <S**2≥0.000
	117 -> 126	0.22897	
	119 -> 125	0.10519	
	119 -> 126	−0.13490	
	120 -> 125	0.24255	
	121 -> 126	0.15604	
	121 -> 127	−0.23771	
	122 -> 127	−0.13954	
	122 -> 128	−0.11262	
	123 -> 128	0.17716	
22	112 -> 124	0.10525	Singlet-A, 6.1447 eV 201.77 nm f = 0.0063 <S**2≥0.000
	113 -> 124	−0.10746	
	114 -> 124	0.31542	
	115 -> 124	0.26422	
	116 -> 124	−0.11814	
	119 -> 126	0.10383	
	120 -> 125	−0.29128	

	120 -> 126	0.34837	
	121 -> 126	0.12390	
23	114 -> 124	−0.18238	Singlet-A, 6.1484 eV 201.65 nm f = 0.0947 <S**2≥0.000
	115 -> 124	−0.14505	
	119 -> 126	0.17306	
	120 -> 125	0.22991	
	120 -> 126	0.55219	
24	117 -> 125	−0.13124	Singlet-A, 6.1771 eV 200.72 nm f = 0.0022 <S**2≥0.000
	118 -> 125	0.62448	
	119 -> 125	0.28416	
25	112 -> 124	0.17334	Singlet-A, 6.2169 eV 199.43 nm f = 0.1866 <S**2≥0.000
	113 -> 124	−0.11065	
	114 ->124	0.45043	
	115 -> 124	−0.19646	
	120 -> 125	0.26942	
	122 -> 127	−0.11567	
	122 -> 128	−0.14503	
	123 -> 128	0.14629	

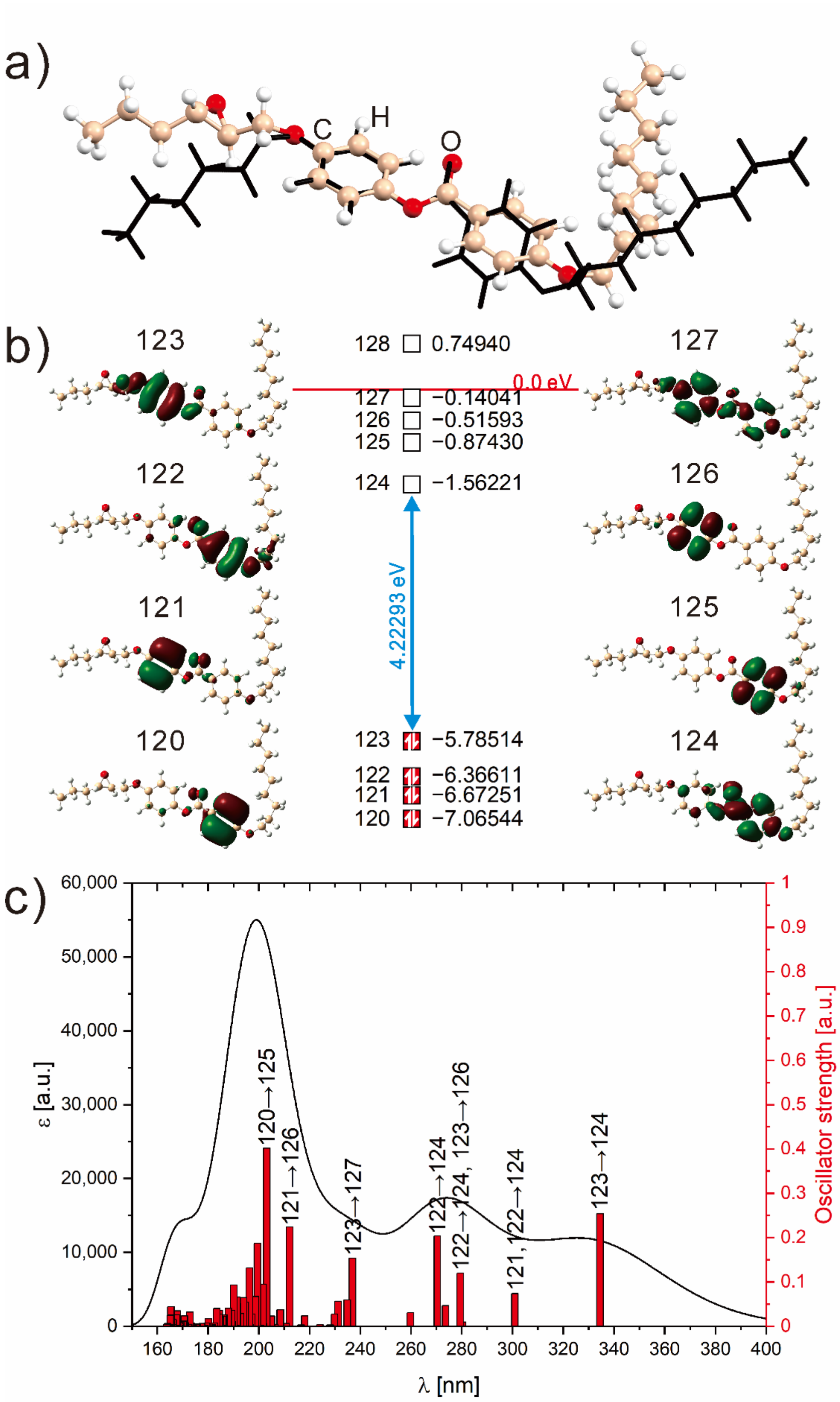

Figure S6. Model of 'planar' EHPDB molecule used for calculation (**a**). Black sticks indicate an overlapped optimized structure of EHPDB. Energy level diagram in eVs with calculated frontier molecular orbitals (**b**). Blue arrow and number correspond to the HOMO-LUMO gap. Calculated UV-Vis spectrum for 'planar' EHPDB molecule (**c**). Red bars indicate electronic transitions with the largest contributions while legend above bars indicate electronic states involved in transition.
